# Field Cycling from 10 nT to 9.4 T: A Flexible Gear Rod Design for Nuclear Spin Relaxation and Hyperpolarization Studies

**DOI:** 10.1021/acsmeasuresciau.5c00131

**Published:** 2025-12-01

**Authors:** Josh P. Peters, Charbel D. Assaf, Mathis Côté, Jan-Bernd Hövener, Andrey N. Pravdivtsev

**Affiliations:** † Section Biomedical Imaging (SBMI), Molecular Imaging North Competence Center (MOINCC), Department of Radiology and Neuroradiology, University Hospital Schleswig-Holstein, Kiel University, Am Botanischen Garten 18, 24118 Kiel, Germany; ‡ Department of Engineering Physics, 5596 Polytechnique Montréal, 2500 Chem. de Polytechnique, Montreal, Quebec H3T 1J4, Canada

**Keywords:** magnetic field cycling, hyperpolarization, relaxation, SABRE, chemical exchange, nuclear magnetic resonance

## Abstract

We present a flexible gear rod-based magnetic field cycling (MFC) system for high-resolution NMR spectrometers. The system enables the transfer of the sample from the NMR *B*_0_ field of 9.4 T to ∼nT and all fields in between within 1 s. A flexible gear rod was essential for reducing the total height to approximately the height required for filling the NMR with liquid helium. Due to its reduced height, it can be installed in average-size NMR laboratories (the height of the NMR with MFC is only 3.32 m). Only off-the-shelf components and 3D-printed parts were used for the system assembly, lowering the costs for replication. An automated shimming procedure for ultralow fields is presented to achieve homogeneous fields of a few nanotesla. The system utility is exemplified by measuring *T*_1_ relaxation dispersion of the most common liquid state hyperpolarization tracer—[1-^13^C]­pyruvate—and magnetic field dependences of signal amplification by reversible exchange, enabling alignment transfer to heteronuclei (SABRE-SHEATH) hyperpolarization of [^15^N]­pyridine. Using the system, we uncovered the exact relaxation dispersion of pyruvate for a standard preclinical dDNP sample composition and provided quantitative estimates for the retained polarization after sample transfer. We modified the observation protocol of SABRE-SHEATH polarization, which, with the high reproducibility of the MFC, provided us with a method to measure the chemical exchange rates of hyperpolarized compounds.

## Graphical abstract

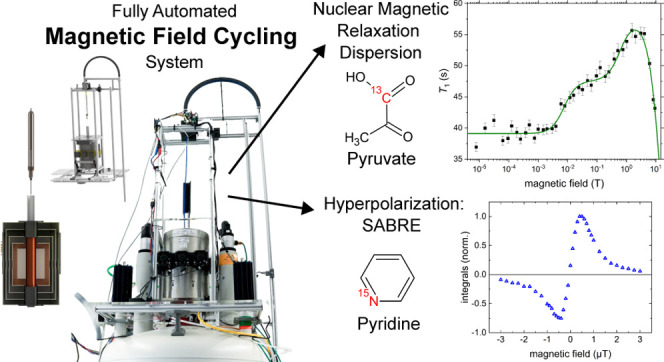

## Introduction

Commercial NMR spectrometers are versatile and powerful tools for molecular analysis in a given *B*_0_ field. Measuring parameters in different fields, however, typically requires several devices or custom-made magnetic field cycling (MFC) systems, which can provide additional information about molecular systems, by measuring *T*_1_, *T*_2_ relaxation times, and diffusion as a function of magnetic field.^1–7^

Varying magnetic fields are essential to extract parameters such as chemical shift anisotropy (CSA), molecular correlation times, *τ*_c_, and chemical exchange rates.^1–10^ Likewise, relaxation is particularly important for hyperpolarization methods, such as chemically induced dynamic nuclear polarization (CIDNP),^11–14^ parahydrogen-induced polarization (PHIP),^15–18^ Overhauser dynamic nuclear polarization (ODNP),^19,20^ and signal amplification by reversible exchange (SABRE).^21–23^ These methods are strongly magnetic-field dependent, and MFC-based studies can reveal properties such as free radical *g*-factors, signs and magnitudes of hyperfine couplings, and nuclear spin–spin interactions.^24^ Moreover, MFC is essential for optimizing polarization transfer in these techniques.^25–27^

Various mechanical designs for MFC systems have been developed, including pneumatic actuators,^28^ rope-and-pulley systems,^29–32^ timing belts,^14,20,33^ gear rods,^34,35^ and robotic arms.^36,37^ Each design has its trade-offs. For instance, rope-and-pulley systems are compact but relatively slow and often rely on gravity unless pneumatics are used.^38^ Gear-rod systems are relatively simple but demand ample vertical clearance due to long gear rods on top of the high-resolution NMR.^34^ Systems with powerful floor-mounted motors and timing belts can introduce mechanical instability and vibration, often necessitating the elevation of the NMR magnet and its fixation to the wall, which increases susceptibility to mechanical vibrations.^20^ There are MFC systems that change the current in electromagnets to vary the field instead of physically moving the sample.^2^ These systems allow much faster field cycling; however, they offer limited spectral resolution and relatively low sensitivity compared to modern high-resolution NMR systems.^3,39–41^

The effect of the field on the relaxation of selected molecules is remarkable. For example, we and others have observed unexpectedly rapid relaxation for specific molecules, such as nicotinamide,^42,43^ succinate,^44–46^ and fumarate,^46^ under particular conditions like low pH or low magnetic fields. In these cases, the “retained” polarization (at the time of detection following hyperpolarization) was significantly lower than expected, prompting MFC to study the underlying mechanisms. For example, paramagnetic impurities are a known cause of rapid relaxation at low magnetic fields,^41^ even for long-lived spin states,^47,48^ and the impact of such impurities can be examined and quantified using MFC. For this reason, MFC was already used to study relaxation as a function of magnetic field (nuclear magnetic relaxation dispersion, NMRD) to estimate polarization losses during the transport of hyperpolarized materials.^31,40^

However, the current implementations of MFC setups are either not commercially available, limited in performance or improperly sized (e.g., ceiling height, throughput, field range, and automation). In addition, comprehensive MFC experiments require many individual experiments, so that the entire experiment becomes so long and complex that it is often not conducted, even though it is necessary. A suitable example is [1-^13^C]­pyruvate, a very promising candidate for use as a hyperpolarized contrast agent for measuring metabolism.^49,50^ Here, considerable research efforts continue to be spent, but the detailed *T*_1_ and *T*_2_ dispersion using thermally polarized samples at the relevant fields from 10^–6^ T to 10 T were never measured. Still, some values in a few fields using hyperpolarized pyruvate in various solvents and of different isotopomers were reported.^40,51–54^

To address these needs, we developed our own MFC system, which had to meet several requirements: be no more than slightly higher than required for the NMR system itself, enable rapid automated shuttling, and provide homogeneous low to ultralow magnetic fields. The system was integrated into a standard 400 MHz high-resolution NMR spectrometer. It enabled highly reproducible MFC experiments across 9 orders of magnitude of magnetic field by moving an NMR tube between the low and observation fields in less than 1 s. We are utilizing a flexible gear rack to overcome spatial limitations such as a low ceiling height (Figure 1). An automated ultralow field shimming routine provided a homogeneity of a few nanotesla for the application field.

**Figure 1:**
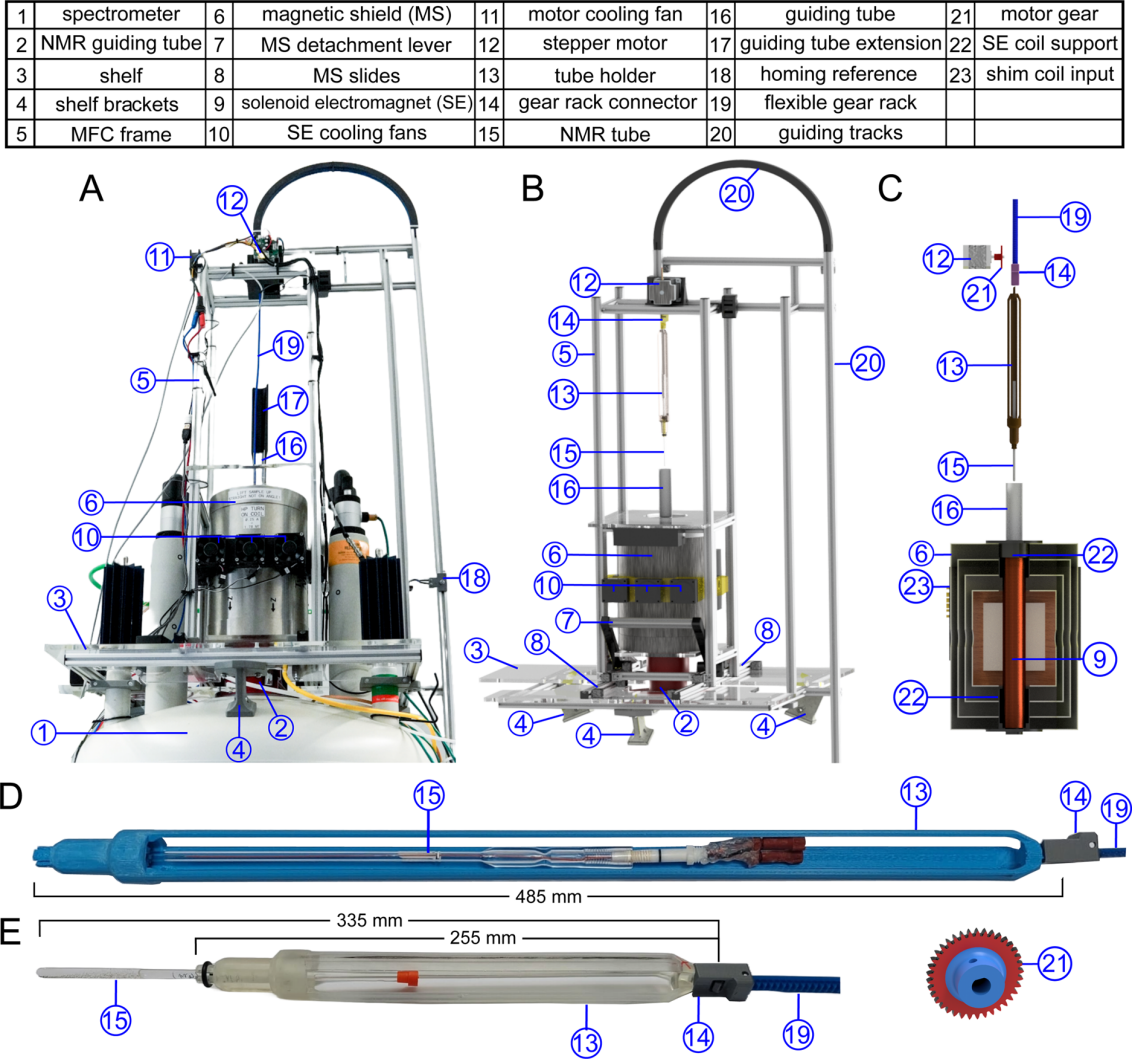
Magnetic field cycling (MFC) system. Photo (A), 3D rendering of the complete setup (B), cross section of the magnetic field shield (C), and tube holder (13) for standard (E) and high-pressure (D) NMR tubes. The base plate of the setup (3) was connected to the lifting lugs of the spectrometer in three positions (4). Guiding tracks (20) were used to connect a four-rung frame (5) to the plate, housing the magnetic shield (6) and a stepper motor on top (12). Magnetic shield (MS) detachment lever (7) was used to place (or remove) the MS on the spectrometer guiding tube (red tube) with its pins. Three fans (10) were used to provide sufficient airflow through dedicated ducts for thermal stability of the MS (6) and SE (9), and an additional fan (11) was used to cool the stepper motor (12). NMR tubes (15) were held in a tube holder (13), and fastened to a flexible gear rod with a custom quick connector (14). This gear rod was inserted into the curved guiding track (20) and connected to the motor gear (21). In an experiment, the shuttle with the NMR tube travels through the three-component guiding tube system (2, 16, 17). As it enters the magnetic shield (6), the NMR tube passes through SE (9), which was aligned with the MS through dedicated supports (22). The homogeneity of the field inside the MS was adjusted using the built-in shim coils driven by external power supplies connected with sockets (23). Note that in D, the NMR tube is not placed in its working position, while in E, the NMR tube is placed in its working position.

We exemplified the power of the setup by measuring the *T*_1_ relaxation dispersion for [1-^13^C]­pyruvate in the solution after dissolution DNP experiments and used this data to estimate the polarization loss during transfer to the detection site. In addition, we demonstrated hyperpolarizing [^15^N]­pyridine using signal amplification by reversible exchange in shield enables alignment transfer to heteronuclei (SABRE-SHEATH)^55,56^ and measuring the chemical exchange rate of pyridine with Ir-complex using hyperpolarized signals.

## Results and Discussion

### Automated NMR Magnetic Field Cycling

A general-purpose MFC system (Figure 1) was constructed from commercial and custom-made aluminum and 3D printed components (see the complete list in Table S1, Supporting Information) and mounted on a spectrometer on a dedicated MFC frame. The device was securely attached to the lifting lugs of the NMR magnet using three brackets, which also provided stable alignment between the NMR probe and the MFC assembly.

The mainframe of the device was a 24 cm × 24 cm × 100 cm (left, right, height) cuboid constructed from four aluminum T-slot profiles (20 mm × 20 mm, 98 cm in length). A 30 cm tall magnetic shield (MS) was installed on a 24 × 24 cm^2^, 1 cm thick acrylic plate at the bottom of the device (first level). On the second level above the MS (height of 53 cm), the bore of the MS is open. This is where the sample is connected to a flexible gear rack for shuttling. A second plexiglass plate separates the two levels, providing additional mechanical support and electrical isolation. A stepper motor on top of the structure was used to drive the flexible gear rod; curved guiding tracks were used to direct the rod on a 20 cm-radius half-circle to reduce the height of the MFC.

The highest point of the NMR spectrometer in standard configuration was 247 cm, with the liquid helium filling port at 242 cm. For refilling helium, an additional headspace of 74 cm is required (without any further clearance), resulting in a minimum ceiling height of 316 cm for the regular NMR operation. Our MFC system reaches a height of 332 cm, i.e., only 16 cm more than the minimum installation requirement. The total distance from the NMR magnet *B*_0_ to the beginning of the guiding tube is 120.1 cm. This setup allowed us to shuttle a 56 cm-long NMR tube assembly (for high-pressure NMR tubes, see below); if only regular NMR tubes are required, the NMR tube assembly can be as small as 33 cm, reducing the total height of the NMR with MFC by 23 cm to 309 cm, around 7 cm below the minimum required ceiling height. This MFC system should fit any room suitable to accommodate an NMR spectrometer.

If a solid gear rod MFC system were to be used, a total of 120.1 cm (for movement) plus the size of the shuttle and a stepper motor on top of the MS is needed. This results in at least 392 cm total height to accommodate the longer shuttle, exceeding the minimum required ceiling height by 76 cm (and our ceiling height by 45 cm). This showcases the need for the flexible rod system presented here.

The complete shuttle consists of four parts, from bottom to top: the tube holder (13), the NMR tube (15), the gear rack connector (14), and the gear rack (19). The bottom part of the tube holder (Figure 1D,E) was constructed similarly to the manufacturer’s NMR spinner and holds the NMR tube. The extension (middle part) consists of three guiding rods that align the shuttle in the guiding tube; its length is dictated by the length of the NMR tube in use, possibly including tubings needed for SABRE-based experiments. A custom 3D-printed push-click connector was used on top, enabling rapid exchange of the NMR tube assembly. All shuttling components are lightweight: tube holder (short, 27.8 g; long, 47.8 g), flexible gear rack (57.7 g), standard NMR tube (3 g), high-pressure NMR tube with tubing (20 g), and gear rack connector (4 g). This resulted in a total shuttling weight of 92.5 g for relaxation experiments and 129.5 g for experiments involving gases, such as SABRE-SHEATH. However, as discussed below for the current design, friction is more restrictive for the shuttling speeds than the mass.

As the MS, we chose a compact 4-layer μ-metal shield with integrated shimming coils and a custom axial bore of 54 mm (increased from 25 mm, based on MS-1L, Twinleaf). Inside the MS, a hollow anodized aluminum cylinder (outer diameter 30 mm, inner diameter 25.4 mm, and length 420 mm) served to guide the NMR tube shuttle and to hold a resistive solenoid electromagnet (SE) (length 300 mm, six layers, 273 turns each, 1 mm isolated copper wire, *R* = 4.36 Ω).

The SE and MS units were equipped with three cooling fans (Figure 1(10)). For ultralow-field experiments, the currents are very small, so active cooling is not required — for example, in SABRE-SHEATH experiments (discussed below), only about 3 mA was used, producing merely 0.4 μW of heat. In contrast, relaxometry studies required magnetic fields of several millitesla, corresponding to currents above 1 A and dissipating more than 4 W of power. To prevent sample heating under these conditions, the cooling fans were implemented. During standard shuttling experiments, the sample was typically equilibrated to room temperature, and no temperature-induced chemical shift variations were observed. However, in specific cases, such as SABRE or PHIP,^56–58^ where reaction rates are temperature-dependent, dedicated sample temperature control must be implemented.

A complete mechatronic solution (PD60-3-1260-TMCL, Reichelt elektronik) comprising of a stepper control module (TMCM-1260) and a stepper motor (NEMA24) was used to drive a flexible gear rack to shuttle the sample between the high and low fields. The flexible gear rack slid into the bent guiding track. At the end of the guiding track, a physical switch was used as the “homing reference” and to automate calibration of the top position (0 μsteps, 0%, outside). The complete construction, while sturdy, is lightweight (below 30 kg including 8 kg from MS-1L) and easy to assemble. No modification was necessary to the spectrometer, assuring normal operation without interference from the MFC hardware. The main construction with the magnetic shield can be removed by loosening four screws and sliding it from the platform if needed.

Using this system, we achieved shuttling between the NMR and MS/SW chambers within 1354 ms, covering a distance of over 1.2 m, and returning in 1018 ms; all intermediate positions were reached even faster. The acceleration for downward motion was 6552 mm/s^2^ (180,000 μsteps/s^2^), with a maximum velocity of 1747.2 mm/s (48,000 μsteps/s); half the maximum acceleration and twice the maximum velocity were used for motion in the opposite direction, due to the different state of the flexible rod during the transfer. The flexible design causes the rod to be under tension during upward motion, resulting in a higher allowed acceleration. During downward motion, friction from the tube holder resists movement and may lead to slight rod bending if too high accelerations are used. Currently for speed, the main limitation is the high friction experienced by the flexible rod within the curved guiding track. Because motor torque decreases with increasing speed while friction rises, employing a motor with twice the power would not provide a significant advantage. A more effective improvement would be to redesign the guiding track to further reduce friction.

The present MFC setup can, in principle, shuttle at least twice as fast without compromising spectral resolution. However, such high-speed settings were not routinely used, as friction-induced resistance occasionally caused stalls that interrupted ongoing experiments. To ensure stable multiday operation, slower but more reliable motion settings were therefore employed. With these optimized parameters and finely tuned 3D-printed components (discussed in the Supporting Information), over 50 km of cumulative sample shuttling distance was achieved without any material failure or stalling.

### Magnetic Field Cycling: Electronics and Software

To ensure reproducible MFC experiments, it was essential to synchronize all components of the system, which include the 400 MHz NMR spectrometer, the shuttling mechanism, the power supply for the fans used to dissipate heat, the power supplies for the SE and the shimming unit of MS and, when performing SABRE or PHIP experiments, a valve system for controlling parahydrogen (pH_2_) delivery.

We developed software that communicated with both the spectrometer and the MFC control system (motor control and integration of all electronic components, Figure 2) to accurately synchronize sample motion with pulse program (PP) execution. While the main control platform is the NMR spectrometer (Bruker’s TopSpin software), an auxiliary user program (AU program) executes both the PP and a Python script (communications slave, CS) to interface between the spectrometer and the MFC software.

**Figure 2:**
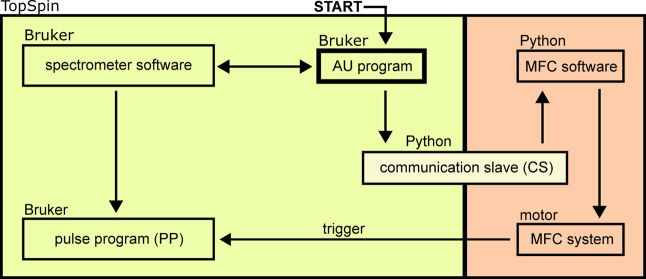
Diagram of the communication protocol for controlling the spectrometer and the MFC. An AU program plays a central role in the distribution of the tasks and sets up the spectrometer software to run the pulse program (PP) in the desired format. The PP then waits for one or more triggers to conduct the desired excitation and acquisition profile. After setting up the PP, an intermediate Python script (communication slave, CS) is called to communicate with the MFC software and hand over the parameters needed for operation, such as shuttling duration arrays, desired field arrays, and more. The MFC software has a motor program corresponding to every NMR spectrometer PP, which is dynamically loaded and executed. The motor is controlled and constantly monitored. When necessary, an output from the motor can be used to trigger the NMR console and, thus, the PP. When an acquisition is completed, it may be repeated with the same variables for averaging or different variables to complete desired pseudo-2D experiments.

Before starting an experiment, the MFC software starts a transmission control protocol (TCP) server to receive commands from the CS, which are used to set parameters such as the desired shuttling field, shuttling duration, position, or any other set of variables.

Typically, at least one parameter is varied during the experiment. For example, in relaxometry, the time (variable duration parameter, vd) at the low magnetic field (application field) is varied. Such an experiment allows recording data as a pseudo-2D experiment (in TopSpin). To accommodate such 2D experiments, up to three arrays and 17 variables can be handed from the AU to the MFC software.

A digital output of the motor was connected to a trigger input on the NMR console to synchronize the motor with the PP. Triggered steps in the PP ensured accurate timing, especially when running parts of the PP before the shuttling. When working with SABRE, it was possible to use the trigger outputs controlled by the PP to switch the valves for pH_2_ bubbling.

The MFC software was designed to work modularly: while the base software consists of motor control scripts, standard operation procedures, and many additional modules, the user can focus on programming dedicated motor program (MP)­files, which have an easy programming language. Each PP has a dedicated MP file, which is dynamically loaded by the MFC software once the corresponding PP is launched. This ensures easy adjustability and maximum flexibility while keeping the MP files lean.

Several additional scripts were implemented, which convert, for example, a desired magnetic field into position and SE current using field-to-position and field-to-coil-current lookup tables. Using this approach, sampling of the field from a few nT to *B*_0_ (9.4 T) is easily implemented using field and vd arrays. For example, this approach enabled NMRD measurement without intervention. The MFC software uses a serial connection to the motor and constantly monitors the motor’s actions, checking for errors such as deviations during the movement. If abnormalities are detected, the sequence is halted to wait for operator input.

### Risk Assessment and Solutions

It is reassuring that the system has minimal potential to cause damage to the NMR due to its flexible rod and mechanical design features. In the unlikely event of a malfunction during insertion (e.g., if the system attempts to move beyond its intended range), the gear rod will simply curl up on top of the spectrometer until it is fully extruded, at which point the motor spins freely. During extraction, the sample may be lifted completely out of the spectrometer until it reaches the lower motor mount, where it could cause minor damage to the MFC system, but not to the spectrometer itself. Considering the low 3D printing costs, such damage would be insignificant.

Both scenarios are highly improbable because the motor’s on-board microcontroller includes soft stops, predefined boundaries beyond which the motor cannot operate. Additionally, if the motor encoder deviates by more than 3 mm from the motor’s set position, the system automatically enters limb mode and remains locked until the operator manually resets it. For further safety, the motor’s enable pin is connected to an emergency-stop button placed at the operator’s desk, allowing the motor and MFC system to be shut down instantly if all other safeguards fail.

So far, only encoder deviation has ever been triggered, when acceleration or velocity parameters were set beyond the standard operational limits. As mentioned above, when such abnormalities are detected by the MFC software, the sequence halts automatically and awaits operator input. No motor or NMR actions can continue until the system is inspected and the “homing reference” is recalibrated, after which the experiment can be safely resumed.

### Magnetic Field Profile

The magnetic field was measured along the *z*-axis automatically using a teslameter (F71, Lake Shore Cryotronics) with an axial hall sensor (FP-2X-250-AS15, Lake Shore Cryotronics) across the sample shuttling distance, starting with the outside position (0%, 0 μsteps) and finishing at the isocenter of the NMR *B*_0_ field (100%, 33,000 μsteps) in 2000 individual steps. These measurements were repeated with 31 SE currents between −1.5 and 1.5 A (Figure 3). The magnetic field profile was obtained this way to generate a field-to-position lookup table and corresponding script. Additionally, the magnetic field probe was placed at position 3.34% in the middle of the MS and SE, where the SE current was varied in 1041 steps from 0 to 3 A (about 39.2 W of SE power dissipation). This calibration was used for a field-to-current lookup table. When a specific low magnetic field is desired, a script is executed and dictates the required current and position for shuttling to access this field using the power supply and the motor.

**Figure 3:**
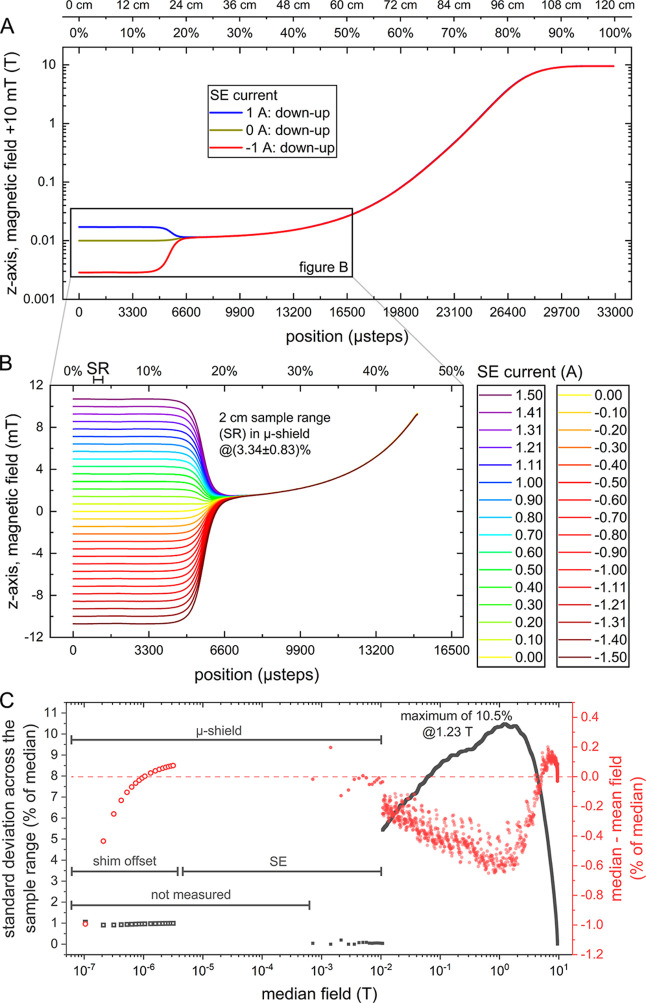
Magnetic field profiles and homogeneity. Mean magnetic field as a function of the position across the entire shuttling range (A) and a subsection of the profile influenced by the SE inside MS (B). Assessment of magnetic field homogeneity across the sample range (SR) of 2 cm (C). (A) A plateau of the magnetic field was observed close to the sweet spot of *B*_0_ field at 33,000 μsteps position, and a steep increase when moving between 15,000 and 28,000 μsteps. Note that the field is given with the +10 mT offset to incorporate negative fields in a log-*y* axis and demonstrate that the field orientation changes when the negative field is applied. (B) The field was homogeneous in at least 4000 μsteps or ∼64 mm (0.0364 mm/μstep). The field to current ratio of the SE was ∼7.1 mT/A. The SR inside the MS and SE sweet spot is indicated at position 3.34%. (C) The standard deviation of the magnetic field across the SR (gray squares) is always below 10.5% of the median field, and the highest at 1.2 T, where the slope of the field curve is the steepest per distance. The deviation of the median field from the mean field is always below 1% (red circles). The highest homogeneity is achieved inside MS/SE unit for fields below 12 mT. The specific field profiles inside MS have not been measured but simulated using the same methods as in Figure 4 (hollow symbols). Note that the exact magnetic field at 0 A of applied current depends on the shimming of the MS, but may reach a few nT.

A zero-field crossing was observable when negative SE currents were used; hence, positive currents were chosen for most future experiments. For fields above 12 mT requiring more than 1.65 A, the MFC system was designed to go to the respective position at the stray field of NMR.

Using the stray *B*_*z*_ field is convenient, but it causes field variations across the sample because the stray field varies along the *z*-axis. This results in (a) inhomogeneities (quantified by standard deviation, SD) and (b) a mismatch between the set and experienced average field (Figure 3C), since we chose to position the center of the sample at the desired field. This, of course, may be different from the mean field if the field is nonlinear across the sample. We investigated this effect for a 2 cm sample range and found a maximum difference of the mean and the set field of 0.5% in the stray field.

For fields below 12 mT, the MFC will instead go to position 3.34%, and the power supplies of SE and MS will set the required field. Hence, the shuttling duration and polarization losses during sample transfer to fields above 12 mT will be lower, aiding in sensitivity when working with quickly relaxing molecules. However, the homogeneity of the SE field is better compared to the field in the NMR bore and was thus preferred for <12 mT.

For fields below a few μT, a current of <1 mA would be required for the SE, which was not possible with the high current PSU used to generate fields of up to 12 mT. Therefore, for lower fields, the *z*-shim coil of the μ-shield was used to create smaller fields of (6.2 ± 0.5) nT to about (3.16 ± 0.03) μT by using an offset current on top of the optimized shim values (see below). To measure such low fields, we used a magnetoresistive vector magnetometer (VMR, Twinleaf). These field values across the sample range for offsets >0 have been simulated using the methods presented in Figure 4.

**Figure 4:**
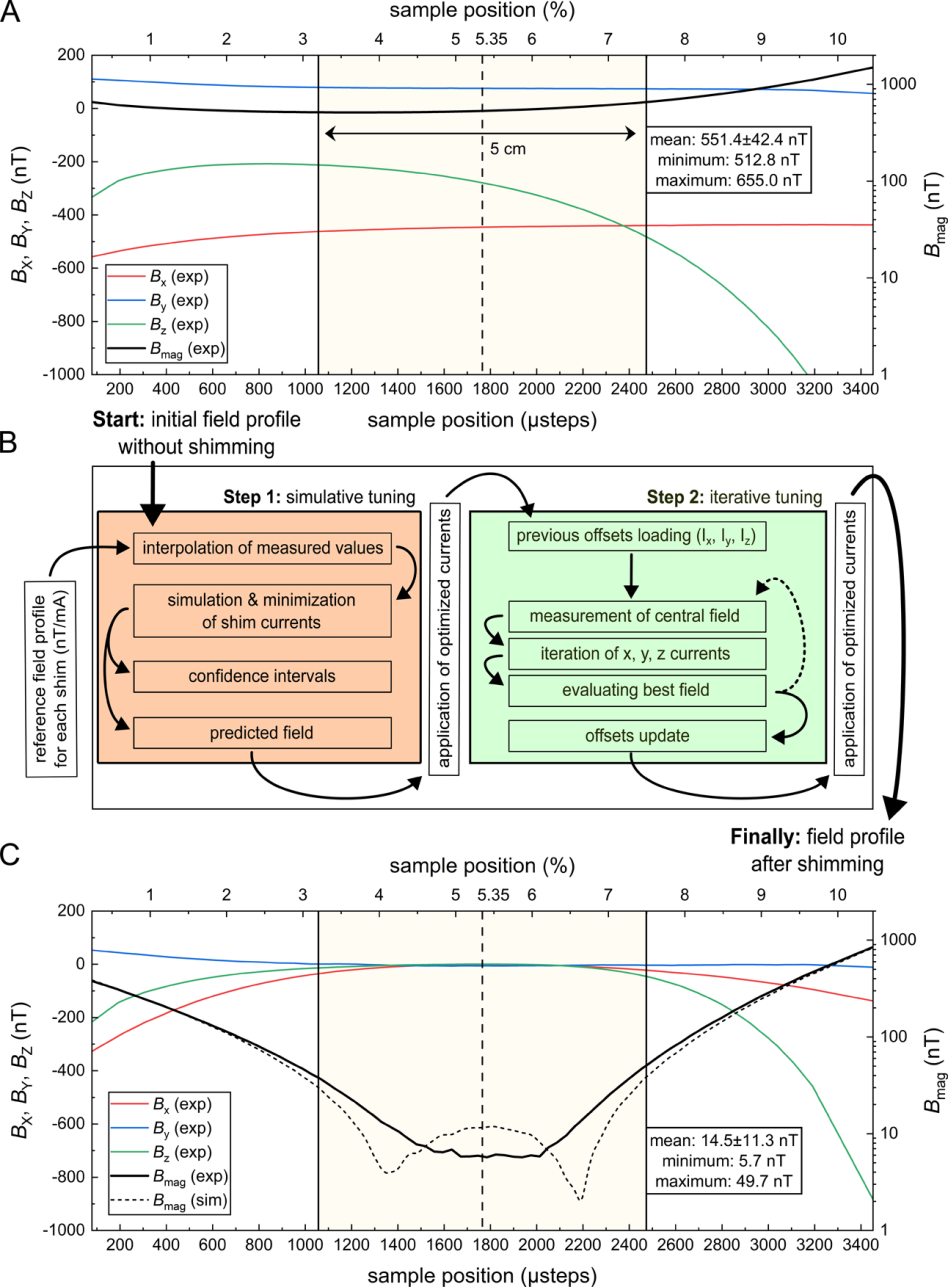
Initial magnetic field profile inside the μ-shield without shimming (A), schematic of the shimming procedure (B), and comparison between the simulated magnetic field (dashed) and the measured magnetic field (straight line) after applying the optimization (C). (A, C) The measured magnetic field components (*X*, *Y*, *Z*), respectively represented in red, blue, and green, are linked with the left vertical axis. The measured magnetic field magnitude corresponds to the black curve and relates to the right axis (logarithmic scale). The 5 cm sample region is highlighted in light yellow. (A) The field was (551.4 ± 42.4) nT, with most of the contribution in the *XY*-plane. Still, a relative standard deviation of about 8% reveals a relatively homogeneous field. (B) The shimming optimization algorithm can be grouped into two distinct processes: the simulative tuning and the iterative tuning. A reference magnetic field profile per unit current, ***μ***_coil_(*z*), was first acquired for each of the 9 shim coils, along with the background field ***B***_b_(*z*). The simulative tuning determined the current for each shim coil that minimized the expected magnetic field profile, calculated as ***B***_sim_(*z*) = ***B***_b_(*z*) + ∑*I*_coil_· ***μ***_coil_(*z*). The iterative tuning then adjusted *I*_coil_ for the *X*, *Y* and *Z* shim coils until the magnetic field at *z*_sample_ was minimized. The data and all steps are detailed in methods and Supporting Information. (C) The applied optimization process reduced the field to (14.5 ± 11.3) nT. Note that the dotted field magnitude curve was simulated and created before the iterative shimming. However, the measured field components and magnitude are the results from the whole optimization process, including the iterative shimming. Still, both curves are somewhat similar in appearance, while the differences are exaggerated by the logarithmic scale. Details: The final values for the shimming were (in mA) *X* = −1.98, *Y* = 6.55, *Z* = −3.80, d*Y*/d*x* = −6.97, d*Z*/d*x* = −0.72, d*Z*/d*y* = 2.82, d*Y*/d*y* = −6.85, d*Z*/d*z* = 15.65, and d^2^ *Z*/d*z* ^2^ = 10.44.

### Ultralow Field Shimming

An automated shimming procedure has been developed to improve the homogeneity and amplitude of the field inside the μ-shield, since reliable fields between 0 and a few hundred nanotesla are regularly needed for the SABRE experiments. The μ-shield allows for nine different shim coils: *X*, *Y*, *Z*, d*Y*/d*x*, d*Z*/d*x*, d*Z*/d*y*, d*Y*/d*y*, d*Z*/d*z*, d^2^ *Z*/d^2^ *z*.

Each of these shim coils was characterized in order to assess their effect on the magnetic field along the *z*-axis. This was done by measuring the magnetic field vector ***B***_coil_(*z*,*I*) = {*B*_coil*,x*_(*z*,*I*),*B*_coil,*y*_(*z*,*I*),*B*_coil,*z*_(*z*,*I*)} produced by each shim coil individually, each with currents *I* of +20 mA and −20 mA. For each shim coil and magnetic field component, 200 uniformly distributed points were sampled throughout the whole μ-shield length, for a total of 36.4 cm. The magnetic field per unit current was then obtained with $\mu_{coil}=\frac{\Delta B_{coil}}{\Delta I}$ , which has units of nT/mA.

To apply the shimming, the background field ***B***_b_(*z*) was recorded along the desired positions (e.g., 2–5 cm, the length of the sample inside the NMR tube). Then, the prerecorded field profiles ***μ***_coil_(*z*) were used for a minimization algorithm to find the currents for each shim coil that reduce ***B*** (*z*) as close to zero as possible. We found that optimizing all nine shim coils yielded the lowest and most homogeneous results.

Additionally, a simple iteration of three *X*, *Y*, *Z* shim currents was used to reduce the field in the middle of the sample (*z*_sample_) even further . Then, the desired field magnitude *B*_SABRE_(*z*) was reached by only adjusting the *Z*-shim coil. For example, in one experiment, the background field was measured at *B*_b_(*l* = 5 cm) = (551.4 ± 42.4) nT along 5 cm around *z*_sample_ = 5.35%, the position found best suited (Figure 4A). Simulation yielded a field *B*_sim_(*l* = 5 cm) = (10.9 ± 7.2) nT, and experiment found *B*_exp,1_(*l* = 5 cm) = (20.1 ± 11.3) nT. An iterative variation of the *X*-, *Y*-, and *Z*-shim coil currents reduced the field further to *B*_exp,2_(*z*_sample_) = 1.28 nT, and *B*_exp,2_(*l* = 5 cm) = (14.5 ± 11.3) nT with a maximum of 49.7 nT and a minimum field of 5.7 nT (Figure 4B).

### MFC Spectra

To study NMRD, the sample must first be magnetized at the *B*_0_ field and then cycled to lower fields to assess the relaxation rate. X-nuclei typically relax much more slowly than protons; for example, in pyruvate, methyl, *T*_1_^1H^ = 4.9 s while 1-^13^C, *T*_1_^13C^ = 45.5 s. To take advantage of the faster ^1^H relaxation and its higher polarization, we used polarization transfer from ^1^H to ^13^C via the INEPT pulse sequence. INEPT yielded a substantial signal gain of 2.53 for pyruvate, reducing the measurement time required to reach comparable sensitivity with direct ^13^C acquisition by roughly 60-fold. This approach was therefore used for all subsequent pyruvate NMRD experiments. The INEPT spectrum (Figure 5A) was acquired during calibration of the INEPT delays with active decoupling and short repetition time, which caused a slight drift in chemical shift due to temperature variations.

**Figure 5:**
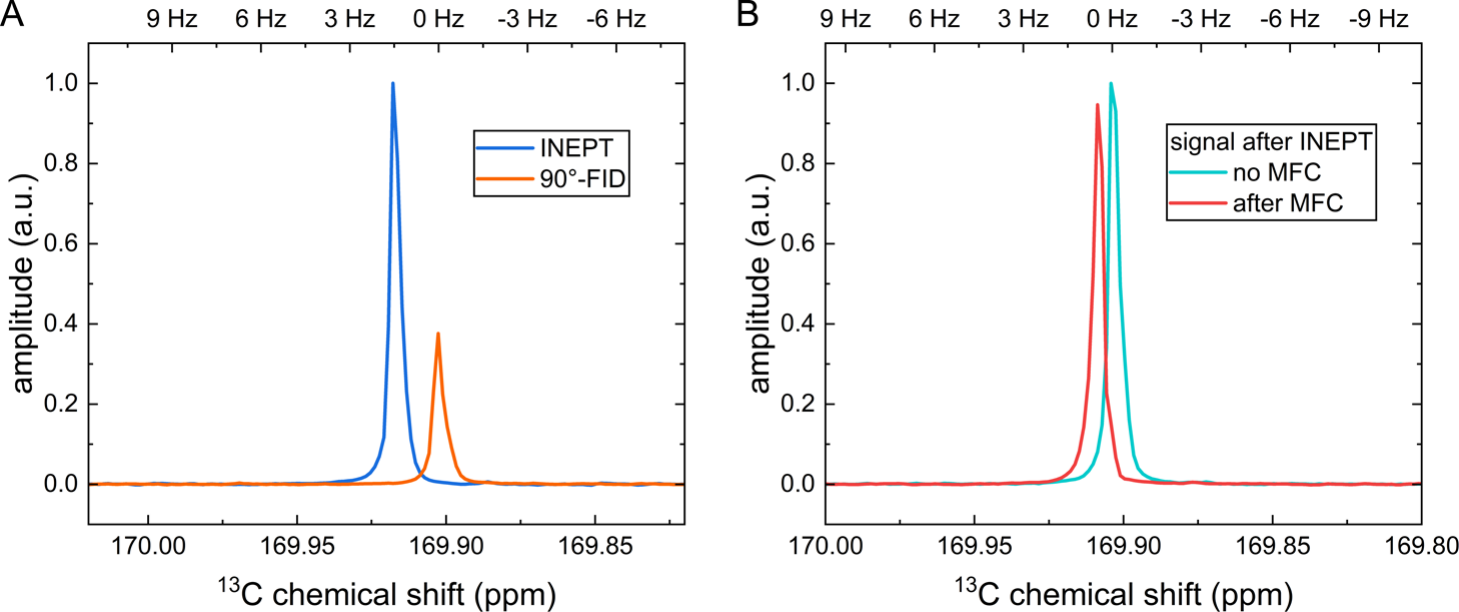
Stationary and MFC ^13^C NMR spectra of [1-^13^C]­pyruvate. (A) Comparison of [1-^13^C]­pyruvate spectra with (blue) and without (orange) ^1^H–^13^C INEPT enhancement. INEPT increased the signal by a factor of 2.53. This enhancement, combined with a shorter relaxation time of ^1^H, T_1_^1H^ = 4.9 s, compared to ^13^C, T_1_^13C^ = 45.5 s, led to a reduction of the measurement time by about 60 times to achieve the same SNR without INEPT. (B) ^1^H–^13^C INEPT enhanced NMR spectra without (stationary, teal) and after MFC in the low magnetic field (red). No distortions in the line shape were visible for the spectrum measured 100 ms after MFC. All spectra were acquired with ^1^H decoupling, and line broadening of 0.2 Hz was applied. Notice the different resonance shift due to temperature swing when performing multiple INEPT sequences with decoupling for INEPT calibration (A), and when performing MFC (B). In panel A, INEPT calibration was performed by repeating 20 consecutive NMR experiments with variable interpulse delays, using a repetition time of approximately 15 s and an acquisition time of 6 s with active ^1^H decoupling (0.37 W). This protocol caused a noticeable temperature fluctuation (∼0.4 K) during the measurements. In both figures, line broadening of 0.2 Hz was applied.

To assess the quality of the spectra after MFC, the spectra before and after shuttling were compared (Figure 5B). A slight loss of signal can be observed after shuttling the sample (position 100%–3.34%–100%–100 ms settling delay–acquisition), due to relaxation. Using a 0 ms settling delay, we observed occasional distortions in ^13^C NMR spectra, whereas with a 100 ms delay, this was not the case; other delays between 0 and 100 ms were not tested. As this delay is sufficiently short compared to all the relaxation times studied here, it was used hereafter. Still, slight distortions are observable in the ^1^H spectra, as they are more sensitive when line broadening is not applied.

### NMRD of [1-^13^C]­pyruvate

To determine the relaxation behavior for the most common dDNP hyperpolarized tracer, [1-^13^C]­pyruvate, we examined its NMRD in a composition typical for dDNP after an actual dissolution DNP experiment containing 36 mM Trizma buffer, 45 mM NaCl, 0.24 mM EDTA, 45 mM NaOH, and 151 μM trityl AH111501 radical in 90% H_2_O and 10% D_2_O (Figure 6). The sample contained 10% D_2_O for locking and shimming. No purification was used after dissolution.

**Figure 6:**
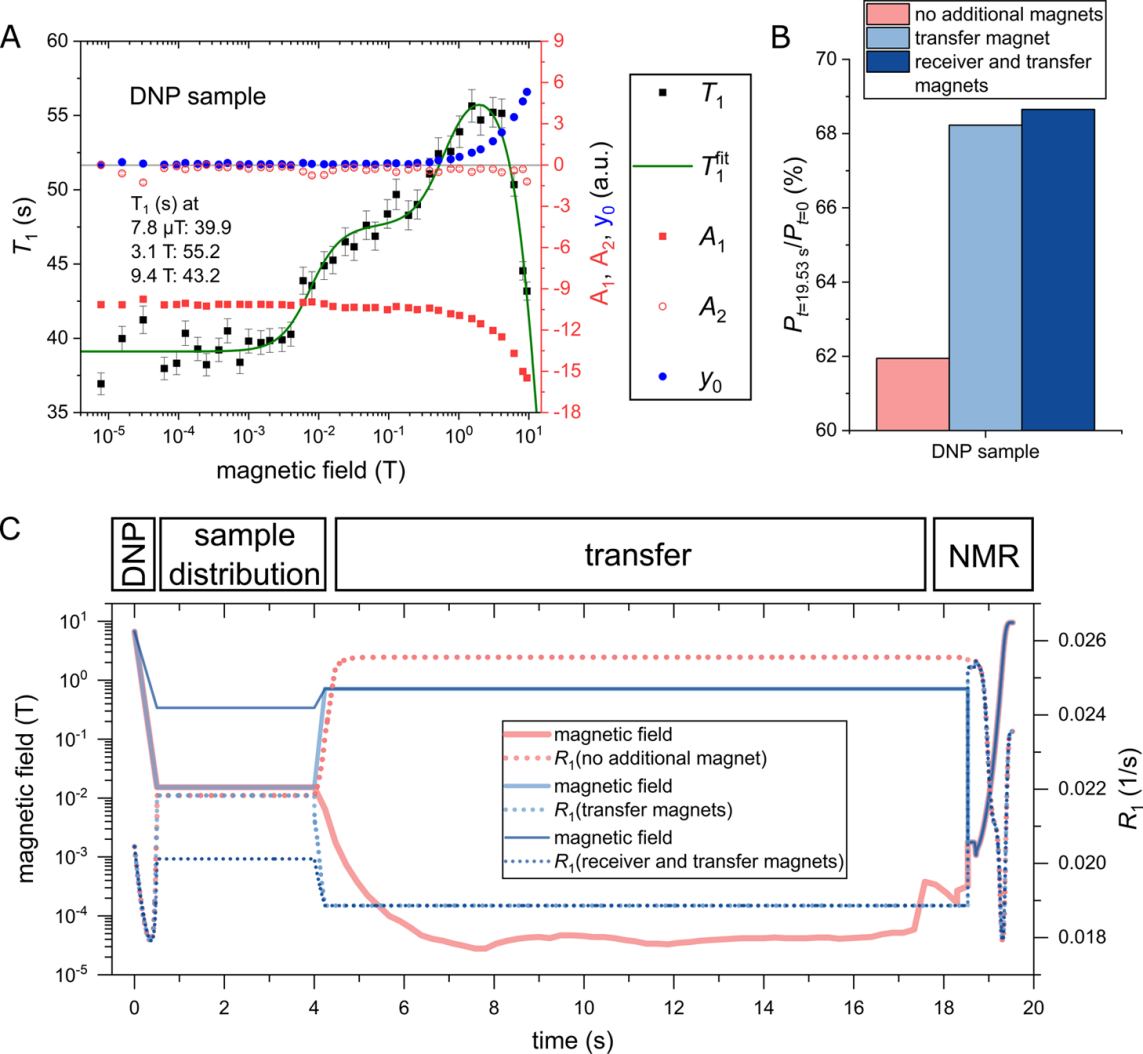
NMRD of [1-^13^C]­pyruvate at fields from 7.8 μT to 9.4 T (A) and impact of [1-^13^C]­pyruvate relaxation and magnetic field on the obtained polarization (B) when transferring from the polarizer to the NMR spectrometer (C). (A) A significant difference between the low-field *T*_1_ and high-field *T*_1_ values is visible, showcasing the need for MFC to quantify the changes in relaxation accurately. The field around 1–3 T gave the longest relaxation time across all samples: the *T*_1_ was always longer than 50 s, in this range, while at Earth’s magnetic fields, it could fall below 30 s. (B) One can see a clear difference between the three transfer cases: no additional magnets (red), transfer magnet (light blue), and receiver + transfer magnets (darker blue). This difference originates from the field-dependent *T*_1_. The dDNP sample would retain only 62.0% without additional magnets. The transfer magnet retained about 6% more of the initial polarization. The benefits of using an additional receiver magnet are limited. Note, the initial polarization value is set to 100%. (C) The three field profiles during transfer from the polarizer to the NMR have been measured to estimate the polarization losses as a result of sample transfer: corresponding relaxation rates are given with dashed lines. **METHODS**: The measured kinetics were fit with a biexponential decay function: $A_{1}e^{-t/\tau_{1}}+A_{2}e^{-t/\tau_{2}}+y_{0}$ . The first value with the largest corresponding amplitude *A*_1_ (red squares) was associated with *T*_1_ (black squares). *y*_0_ (blue circles) is associated with thermal polarization in the given field. The second time constant *τ*_2_ has been shared across all fields to compensate for the observed biexponential decay; note that the contribution is relatively low (amplitude *A*_2_, red circles). The green line corresponds with the fit values of a relaxation model as described in the methods; the fit parameters can be found in Table 1. Sample: 89 mM [1-^13^C]­pyruvate with pH of 7.7 of a standard dDNP sample after actual dissolution DNP experiment containing 36 mM Trizma buffer, 45 mM NaCl, 0.24 mM EDTA, 45 mM NaOH, and 151 μM trityl AH111501rasdical in 90% H_2_O and 10% D_2_O. Pyruvate p*K*_a_ is about 2.5.^59^

For the DNP sample, contaminants such as trityl radical and possible paramagnetic impurities from the superheated dissolution module may be present. However, the low-field *T*_1_ of 40 s was still much longer when compared to more quickly relaxing hyperpolarized tracers,^42–46^ showcasing why pyruvate performs robustly in standard dDNP experiments: it does not require elevated magnetic fields for transport and is relatively resilient to stable radicals. The maximum *T*_1_ was reached at 3.1 T with 55.2 s and dropped to 43.2 s at the highest accessible field of 9.4 T.

### [1-^13^C]­pyruvate Polarization Losses During Transfer

Using the relaxation data obtained with the MFC, we calculated the polarization losses during the transfer from the pyruvate polarizer to the measurement site. To determine the relaxation profile, we measured the magnetic field profile along the transfer path. In our case, it was from the dDNP to the 9.4 T NMR system. The exact timing of the sample transfer—including dissolution, distribution into a smaller tube, actual transport, and insertion into the NMR spectrometer—was estimated, and the total duration was set to 19.5 s, as in our typical experiments for the transfer distance of 16.8 m. This yielded the magnetic field profile experienced by the sample during the transfer, shown in Figure 6C. Based on this profile, the polarization loss (from an initial value set to 100%) was calculated for three different scenarios: (i) no additional magnets, (ii) transfer within a dedicated magnet between the dDNP and NMR systems, and (iii) the dissolution receiver vessel equipped with an additional magnet additional to the one used in (ii).

A clear difference between the no-magnet and transfer-magnet cases can be observed: 6.3% more of the initial polarization are retained for the dDNP sample, which is 10.1% more than without transfer magnets. The difference between cases (ii) and (iii) was only 0.6%. However, it may provide a bigger relevance for quickly relaxing molecules such as [1-^15^N]­nicotinamide.^42,43^

For the dDNP sample, no transfer magnet was used after dDNP, and the observed polarization was 27.3%. Using the calculated polarization losses of 38.1% from the initial value (Figure 6B), the initial value can be estimated to be about 44.1% at the time of dissolution. Therefore, using transfer magnets like in (iii) would have retained 30.3%.

### MFC for SABRE-SHEATH

In SABRE, the target substrate is polarized upon transient interaction of pH_2_ and substrate with Ir-complex (Figure 7A). The optimal polarization transfer field (PTF) varies across substrates due to specific SABRE matching conditions, which are determined by the *J*-couplings and the differences of Larmor frequencies of the hydride and target nuclei.^23,55^ Typically, the polarization in such experiments is most effective at a particular level anticrossing (LAC) field; however, in the case of SABRE, the actual optimal PTF is shifted due to chemical exchange-induced modulations of spin interactions.^60,61^ For ^15^N SABRE-SHEATH, the optimal polarization transfer occurs in the microtesla (μT) field range. To find optimal conditions for SABRE-SHEATH, experiments were previously carried out with automatic MFC or manual shuttling: the exact position of the optimum depends on temperature, deuteration of ligands, impurities, or intentionally added coligands and shuttling magnetic field profile, but the typical range for the maximum was between 0.3 and 0.5 μT^25,62,63^ .

**Figure 7:**
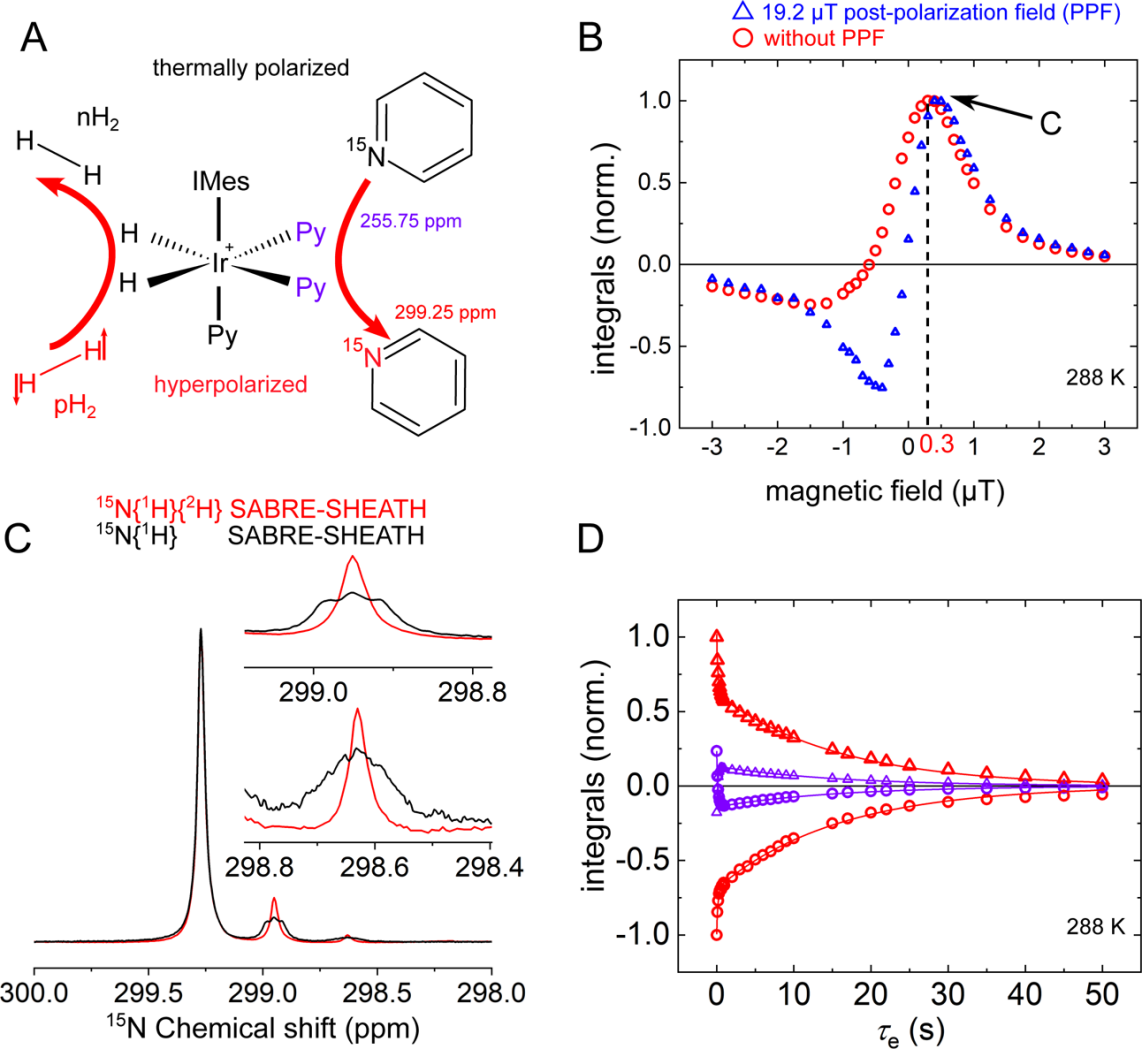
Hyperpolarization of [^15^N]­pyridine with SABRE-SHEATH. (A) Scheme of SABRE-SHEATH hyperpolarization of pyridine, pH_2_, and pyridine exchanges with the IrIMes complex that can result in hyperpolarization of ^15^N. (B) Normalized integrals of ^15^N signals of free pyridine after SABRE-SHEATH as a function of the magnetic field with (blue triangles) and without (red circles) 19.2 μT postpolarization field (PPF). (C) ^15^N­{^1^H}­{^2^H} (red) and ^15^N­{^1^H} (black) SABRE-SHEATH spectra showing polarized free pyridine formed by supplying 8.5 bar of pH_2_ in methanol-d_4_ at 288 K. Note that the pyridine experienced partial deuteration; therefore, three spectra are present: pyridine-h_5_, -d,h_4_, and -d_2_,h_4_. (D) Chemical exchange between free and bound pyridine was measured at high magnetic fields and 288 K after SABRE-SHEATH hyperpolarization using selective polarization inversion of free (triangles) or bound (circles) pyridine. The integrals of free pyridine are red, and those of bound pyridine are violet. Kinetics were fitted with a shared biexponential decay function, resulting in two decay rates of *k* = (5.77 ± 0.19) s^–1^ and *R* = (0.064 ± 0.0008) s^–1^.

To find the optimum parameters for our system, the PTF dependence of ^15^N hyperpolarization was studied by sweeping the field between −3 and +4 μT. The maximum signal for pyridine was observed at a PTF of +0.3–0.4 μT (Figure 7B) in accordance with previous observations.^25,62,63^ The positive PTF is parallel to the *B*_0_, whereas the negative PTF is antiparallel.

We observed that negative PTFs led to reduced polarization, with a zero-polarization crossing at approximately −0.5 μT. For positive PTFs, around +0.5 μT corresponds to the field of maximum polarization. When spin-order transfer during field cycling is neglected (i.e., for instantaneous ideal cycling), the PTF is expected to be antisymmetric with zero polarization at zero field—an effect that has occasionally been observed experimentally.^25^ However, in practice, deviations are common. Similar behavior has been reported for SABRE,^28,64^ for example, and is typically attributed to partial polarization transfer during shuttling through zero or other LAC fields, where ongoing transfer can diminish the overall polarization. Interestingly, such polarization transfer during magnetic field cycling can also be deliberately exploited.^17,65,66^

In principle, polarization transfer during MFC can be quantified using the magnetic field profile and position as a function of time.^67^ However, mitigating undesired slow transfer is often more critical. To suppress such polarization transfer, we repeated the SABRE-SHEATH experiment with an additional magnetic field ramp to +19.2 μT (postpolarization field, PPF) applied after pH_2_ bubbling ceased and before mechanical sample transfer (Figure 7B). This procedure ensured the instantaneous projection of the spin order on the state at a relatively high field away from possible polarization transfer fields. Hence, further sample transfer did not contribute to the polarization redistribution, as the zero-field regime was avoided.

Note the partial H-D exchange on pyridine with methanol (pyridine-methanol exchange), which leads to the resonance corresponding to three isotopomers (Figure 7C). Considering this and previous observation of D-H exchange when deuterated pyridine was used^68,69^ in deuterated methanol (pyridine-H_2_ exchange), and H-D exchange between H_2_ and methanol (H_2_-methanol exchange), we can conclude that protons (or deuterons) of H_2_, methanol, and ortho protons (carbon positions 2 and 6) of pyridine are all loosely connected and experience mutual exchange.

After optimizing SABRE-SHEATH polarization at a low magnetic field, the exchange rates between free and bound pyridine could be measured at high magnetic fields, leveraging high ^15^N polarization. Following SABRE-SHEATH and MFC, a frequency-selective 180° pulse was applied to one form of pyridine (either free or equatorially bound), followed by an evolution delay *τ*_e_ and a 90° hard pulse for detection at high field. This pulse sequence enables tracking of the polarization transfer due to chemical exchange, allowing us to quantify the exchange kinetics in both directions, from free to bound and from bound to free pyridine (Figure 7D). The effective exchange rate constant *k* = (5.77 ± 0.19) s^–1^ was obtained at 288 K. Considering concentrations of bound and free pyridine, the dissociation rate constant was estimated to be *k*_d_ = (9.5 ± 0.3) s^–1^, and the effective association exchange rate to be *k*_a_^′^ = (1 ± 0.03) s^–1^ (see methods and ref. 70 eq. 16 for model-based evaluation of exchange rates), which resides within the error margins of previously measured values using INEPT enhancement.^71^

Note that the sample position used in Figure 6 was about 15 mm above the 5.35% position, which was closer to the middle of MS but not in the middle of the optimized region (5.35%) of the automated shimming procedure. Still, the changes are not substantial for our experiments, as the standard deviation of the magnetic field within the 15 mm higher sample range is only about 17%.

## Conclusion

We demonstrated only some of the use cases of the MFC system, focusing on the hyperpolarization applications. The availability of the NMRD could help one design the most optimal hyperpolarization experiment, such that the critical steps of purification or sample transfer are carried out at the fields with the longest relaxation time. Pyruvate is relatively insensitive to stable paramagnetic impurities when it is in an aqueous buffered solution with EDTA, as in typical dDNP sample compositions. This explains the high polarization values reported for dDNP even when the radical is not filtered from the solution. The fields between 0.1 and 3 T provided the highest ^13^C *T*_1_ of [1-^13^C]­pyruvate; hence, it should be used, when possible, for manipulations of the hyperpolarized pyruvate or during transfer by using a magnetic tunnel or transfer magnet. For example, ^15^N NMRD study of [1-^15^N]­NAM was already done using this setup, showcasing its capability of probing rapid relaxation time of low sensitivity ^15^N nucleus.^43^ Some molecules experience rapid polarization losses at low fields and pH.^42–46^ The investigations of corresponding NMRD could shed light on their relaxation mechanism, how this rapid relaxation can be circumvented, e.g., using the CIDER effect,^43^ and under what conditions the relaxation time reaches a maximum.

SABRE hyperpolarization can be performed at high and low magnetic fields. Hyperpolarization at low magnetic fields typically provides superior hyperpolarization yield because it enables one to accumulate polarization on the free substrate.^25,72,73^ The high-field experiments, however, due to high resolution, allow the study of the chemical interactions.^71,74^ Therefore, combining low and high fields, as was demonstrated here and before,^56^ with exchange rate measurements, can bring about a valuable synergy.

## Methods

### INEPT Enhanced NMRD Experiment

The INEPT sequence^75^ with refocusing was used without phase cycling:

^13^C: -----*τ*-180_X_-90_X_-τ-180_X_-*τ*-90_Y_-(shuttling)-90_X_-FID.

^1^H: 90_X_-*τ*-180_X_-90_Y_-*τ*-180_X_-*τ*------(shuttling)-decoupling.

The optimum INEPT interpulse delay, *τ*, was calibrated for each sample experimentally. Subsequent MFC experiment followed the following sequence: (1) ^1^H thermal relaxation at *B*_0_ for *D*_*B*0_ = 19 s, (2) INEPT sequence to transfer ^1^H polarization to longitudinal polarization of [1-^13^C] of pyruvate, (3) shuttling to desired low-field, either inside NMR bore or in SE with appropriately set current, (4) waiting for ^13^C relaxation at low-field for *D*_LF_, (5) shuttling to *B*_0_ followed by settling delay of 100 ms, (6) ^13^C 90° excitation and signal acquisition with ^1^H decoupling.

### Shimming of μ-Shield

Calibration profiles for each of the 9 shim axes were acquired to obtain the individual dependence of the field (see Supporting Information). Before each shimming operation, a base field profile without activated shims was acquired. Then, all data sets from the individual shim axes and the base field are interpolated using a cubic spline method to ensure overlapping and sufficiently dense data points. A loss function was defined as $L=\sum_{sample}{∥B∥}^{2}+A$ , where $B=B_{b}+\sum I_{coil}\mu_{coil}$ , and *A* = α∑*I*_coil_^2^ as a regularization term especially designed to minimize the final current values (*I*) for each shim. This regularization allowed to optimize for lower currents, which reduced errors between simulation and experiments due to nonlinearity and field drifts. The gradient of the loss function with regard to the currents was also computed for each shim, with the following equation $\frac{dL}{dI_{coil}}=2\underset{sample}{\sum }B\cdot \mu_{coil}+2\alpha I_{coil}$ . The SciPy optimize.minimize method is then used to optimize the 9 shim currents according to the previously defined loss function and gradients. The gradient-based optimization algorithm trust-constr method was chosen, since its reliability and speed are well adapted to this kind of problem. Subsequently, confidence intervals for each of the optimized currents were estimated by computing their overall Hessian matrix. Inverting this matrix can ultimately provide a good estimation of the *t*-value and the standard deviation for each optimized current, with a 95% confidence level.

This simulative tuning was followed by simple iterative tuning method to reduce possible errors from the simulation. More precisely, after moving to the sample’s center position *z*_sample_, *I*_*X*_, *I*_*Y*_ and *I*_*Z*_ can be automatically tuned using small iterations (10–100 μA), and continuous field-probing at this position. Only the three first-order shims were used here since their individual fields are approximately constant throughout the entire sample range (working as offset), therefore, they do not alter the homogeneity. The iterative fine-tuning was stopped when the field magnitude reached below 2 nT at *z*_sample_.

For the SABRE experiment, a postpolarization-field (PPF) was used to avoid loss of negative polarization; for this the SE with a field of about 19.2 μT was used. Since the SE retained some residual magnetization after usage (about 200 nT), the shimming was performed after turning on the SE for a brief duration to saturate this residual field.

### NMRD Fitting

The fitting was conducted using a model that included two inner sphere equations (*R*_IS_) and chemical shift anisotropy (CSA) induced relaxation (*R*_CSA_, eq. 4 in the ESI of ref. 76).

The functions for *R*_IS_ was

1 RIS=(Δω)2τC1+(ωC13τC)2

where Δ*ω* is a fitted coupling constant (not scalar in the traditional sense), *S*_X_ is the spin number of the coupled nucleus, *τ*_C_ is the correlation time characteristic of this interaction, *ω* ^13^_C_ is the Larmor angular frequencies of the coupled ^13^C nucleus.

The function for *R*_CSA_ was

2 RCSA=15(ωC13δCSA)2τCSA1+(ωC13τCSA)2

where *δ*_CSA_ is the CSA of the studied nucleus and *τ*_CSA_ corresponding correlation time.

The NMRD had two elbows at fields below 1 T, which results in the necessity of having two functions like *R*_IS_. The final fitting curve was achieved by fitting the following superposition

3 $$R=R_{IS\text{\_}1}+R_{IS\text{\_}2}+R_{CSA}$$

Possibly, the two Δ*ω* are the consequence of proton exchange at the COOH group and conversion between ketone and geminal diol (hydrate) form. The fitting resulted in too large values for Δ*ω*, unreasonable for typical molecules (Table 1), indicating different original mechanisms, such as dipole–dipole interaction, which has similar field dependency, but Δ*ω* is substituted with the corresponding dipole–dipole term.^4^ Hence, the actual mechanism cannot be deduced solely from NMRD.

**Table 1:** Fitted Parameters of Figure 6

parameter	DNP sample
Δ*ω*_1_ (rad/s)	91.6 ± 8.7
Δ*ω*_2_ (rad/s)	736.9 ± 42.4
*τ*_C1_ (ns)	541.8 ± 110.9
*τ*_C2_ (ns)	6.6 ± 0.7
*δ*_CSA_ (ppm)	46.1 ± 3.0
*τ*_CSA_ (ps)	3.5 ± 0.4

### Relaxation of Hyperpolarization

To estimate the remaining hyperpolarization, we solved the Bloch equation without thermal magnetization, assuming it is much less than the observed polarization value and SNR

4 $$\frac{P\left(T\right)}{P\left(0\right)}=\exp\left(-\int_{0}^{T}\frac{dt}{T_{1}\left(B\left(t\right)\right)}\right)=\exp\left(-R_{1}^{avg}T\right)$$

where *T* is the total time of the sample transfer, *T*_1_(*B*(*t*)) is the relaxation time as a function of magnetic field, which depends on time and *R*_1_^avg^ is the average *T*_1_ relaxation rate constant experienced by the sample during the transfer.

### SABRE-SHEATH Experiment

A typical SABRE-SHEATH experiment to study polarization as a function of the polarization field (*B*_pol_) involved the following steps: (1) before proceeding with and repeating the following steps, the MS was shimmed to reach a homogeneous zero field (17.0 ± 12.7 nT magnetic field magnitude across 3 cm at the SABRE sample position using the automated tuning as described above), (2) thermal relaxation and temperature equilibration at *B*_0_ for *D*_B0_ of 15 s, (3) the NMR tube was shuttled to the isocenter of the MS, where the desired magnetic field was set (ranging from −3 μT to +4 μT); (4) pH_2_ was bubbled through the solution for t_b_ period; (5) optionally a +19.2 μT postpolarization field (PPF) was switched on; (6) shuttling to *B*_0_ followed by a field-cycling settling delay of 100 ms; and (7) ^15^N 90° excitation and signal acquisition with optional ^1^H and/or ^2^H decoupling.

This procedure was repeated either by altering *B*_pol_ and fixing *t*_b_, or by fixing *B*_pol_ and altering *t*_b_.

To obtain the magnetic field sweep of the SABRE experiment, the field was altered by applying an offset to the *Z*-shim axis. The required z-offset was calculated by probing 25 currents between −30 and +30 mA, and followed by a linear fit to extract the magnetic field of 108 nT per mA on the *Z*-shim axis. This slope was used to calculate the required currents for 37 fields between −3000 and +3000 nT.

### SABRE Sample Preparation and pH_2_ Delivery

We conducted SABRE experiments using 50 mM [^15^N]­pyridine (486183, Sigma-Aldrich) and 4 mM of the iridium *N*-heterocyclic carbene complex [Ir­(COD)­(IMes)­Cl] ([Ir], where COD = 1,5-cyclooctadiene and IMes = 1,3-bis­(2,4,6-trimethylphenyl)­imidazole-2-ylidene)^77^ (obtained from University of York), dissolved in methanol-d_4_ (MeOD, Sigma-Aldrich). 92% parahydrogen (pH_2_) was prepared and supplied at 8.5 bar to a 5 mm high-pressure NMR tube (522-PV-7, Wilmad-LabGlass) containing 400 μL of the SABRE solution. The NMR tube was connected to the pH_2_ control unit^78^ via two flexible 1/16″ polytetrafluoroethylene (PTFE) tubes (1528XL, IDEX Health & Science LLC). These tubes were threaded through the cap of the NMR tube and secured using fast glue (Loctite 3090). For the experiments, the NMR tube was inserted into a custom NMR tube carrier (Figure 1D), which was then loaded into the MFC system. This setup ensured that the supply and exhaust lines remained connected throughout the MFC process, allowing for continuous or on-demand pH_2_ bubbling. To avoid mechanical obstruction during shuttling, the PTFE tubes were attached to the quick connector body (top of the shuttle, Figure 1) with slight tension using a cable tie, minimizing the risk of jamming.

### Exchange Rates Obtained with SABRE-SHEATH

After inducing SABRE-SHEATH polarization, the sample was transferred to the high field of 9.4 T for detection. To measure the chemical exchange between free and bound pyridine under SABRE-SHEATH conditions, a frequency-selective inversion pulse followed by a variable delay was introduced before the final read-out 90° broadband excitation pulse. A frequency-selective inversion 180° pulse (Gaus1_180r.1000 shape, 0.0079983 W, 20.79 dB) was applied for 10 ms to either the free or bound pyridine resonance, followed by a variable free evolution interval *τ*_e_ from 0 to 50 s and a 90° hard pulse (75 W, 21.25 μs). The experiment was performed twice: (1) inverting the polarization of free pyridine, and vice versa, (2) inverting the polarization of bound pyridine and monitoring polarization distribution between two sites. Data were fitted using a global fit of a biexponential decay function: *A*_1_ × exp­(−*τ*_e_ × *k*) + *A*_2_ × exp­(−*τ*_*e*_ × *R*) simultaneously for both kinetics. Here *k* is an effective exchange rate and *R* is effective relaxation.^70,71,79^

Considering initial concentrations of [Ir] = 4 mM and [Pyridine] = 50 mM, leading to the concentration of free pyridine of 50–3·4 mM = 38 mM. The dissociation rate constant of pyridine, *k*_d_, and effective association rate constant, *k*_a_^′^, can be then estimated as follows:^70,71^

$$k_{d}=\frac{k}{0.5+\frac{\left[Ir\right]}{\left[freePyridine\right]}}≅k\cdot 1.65$$

and

5 $$k_{a}^{\prime }=\frac{\left[Ir\right]}{\left[freePyridine\right]}k_{d}=\frac{k}{0.5\frac{\left[freePyridine\right]}{\left[Ir\right]}+1}=k/5.75$$

The factor 0.5 is discussed by Salnikov et al., (eq 16)^70^ and its origin lies in the fact that Ir-complex has two equivalent equatorial pyridine ligands.

## Supplementary Material



## Data Availability

All the raw data, CAD files, and developed and used scripts for MFC, including pulse programs and Python scripts, are available on the Zenodo repository https://doi.org/10.5281/zenodo.16760001. In the Supporting Information, no references were used.

## Notes

Additional materials, a detailed explanation of the shimming routine, NMRDs, and a list of the components needed to assemble the MFC system are available online (PDF)
