# Supplementary material for: Field Cycling from 10 nT to 9.4 T: A Flexible Gear Rod Design for Nuclear Spin Relaxation and Hyperpolarization Studies
Source: ACS Meas Sci Au. 2025 Dec 1;6(1):81–95. doi: 10.1021/acsmeasuresciau.5c00131 (PMC12921617; doi:10.1021/acsmeasuresciau.5c00131)
Supplement: Supplementary file 1 [file tg5c00131_si_001.pdf]

Supporting Information for

Field Cycling from 10 nT to 9.4 T: A Flexible Gear Rod Design for Nuclear Spin Relaxation and Hyperpolarization Studies

Josh P. Peters[1*‡], Charbel D. Assaf[1‡], Mathis Côté[1,2], Jan-Bernd Hövener[1], Andrey N. Pravdivtsev[1*]

1. Section Biomedical Imaging (SBMI), Molecular Imaging North Competence Center (MOINCC), Department of Radiology and Neuroradiology, University Hospital Schleswig-Holstein, Kiel University, Am Botanischen Garten 18, 24118 Kiel, Germany

2. Department of Engineering Physics, Polytechnique Montréal, 2500 Chem. de Polytechnique, Montréal, QC H3T 0A3, Canada

‡ equal contribution

* corresponding authors: josh.peters@rad.uni-kiel.de, andrey.pravdivtsev@rad.uni-kiel.de

Contents:

1. Full list of items required for the construction of the MFC setup 3

2. Reference magnetic field profiles for nine shim axes 5

3. Simulation of Z-shim coil current offsets for SABRE experiment 7

4. Examining the effects of mispositioning the magnetometer with respect to the shim calibration 9

5. Details about the *Python* code for magnetic field shimming 11

6. Raw data of Fig. 5: 12

1. Full list of items required for the construction of the MFC setup

Tab. S1: List of materials for MFC setup. The table is divided into five sections:

1. parts for the shelf (**Fig. 1** components 3, 4),
2. parts for the MFC structure (**Fig. 1**, components 5, 7, 8, 20-aluminum sections of the track),
3. parts needed for shuttling the NMR tube (**Fig. 1**, components 11, 12, 13, 14, 17, 18, 19, 20-plastic sections of the track, 21),
4. parts for the solenoid electromagnet (SE) (**Fig. 1** components 9, 10, 16, 22),
5. magnetic shield (MS) (**Fig. 1**, component 6).

Each section contains four columns: number, part name, amount, and vendor. The assembly is available on Zenodo (DOI: 10.5281/zenodo.16760000).

Sec	No.	Name	Amount	Vendor
1 - shelf	1	Aluminium Systemprofil 20x20 Nut 6 (66 cm)	4	Alu-verkauf.de
	2	PLEXIGLAS® XT 10mm klar (80 cm *32 cm)	2	PLATTENZUSCHNITT 24.DE
	3	Generic 3D-printing filament (PETG) 2.85 mm	1600 g	dasfilament.de
	4	Screws, nuts, and rings	20	Frantos.com
2 – MFC structure	1	Alu Systemprofil 20×20 Nut 6 (100 cm)	5	Alu-verkauf.de
	2	Alu Systemprofil 20×20 Nut 6 (140 cm)	1	Alu-verkauf.de
	3	Alu Systemprofil 20×20 Nut 6 (27 cm)	3	Alu-verkauf.de
	4	Alu Systemprofil 20×20 Nut 6 (42 cm)	1	Alu-verkauf.de
	5	Alu Systemprofil 20×20 Nut 6 (9.5 cm)	3	Alu-verkauf.de
	6	Alu Systemprofil 20×20 Nut 6 (6.5 cm)	1	Alu-verkauf.de
	7	Alu Systemprofil 20×20 Nut 6 (21 cm) (Frame)	4	Alu-verkauf.de
	8	Alu Systemprofil 20×20 Nut 6 (31.5 cm) (Frame)	4	Alu-verkauf.de
	9	Alu Systemprofil 20×20 Nut 6 (19.5 cm) (lift)	1	Alu-verkauf.de
	10	Alu Systemprofil 20×20 Nut 6 (74 cm) (rails)	2	Alu-verkauf.de
	11	Alu Systemprofil 20×40 Nut 6 (42 cm) (Stepper-motor stand)	1	Alu-verkauf.de
	12	PLEXIGLAS® XT 10mm klar (38 cm *32 cm)	1	PLATTENZUSCHNITT 24.DE
	13	PLEXIGLAS® XT 10mm klar (25 cm *28 cm)	1	PLATTENZUSCHNITT 24.DE
	14	Screws, nuts, and rings	30	Frantos.com
	15	Angles 20×20	6	Alu-verkauf.de
3 – shuttling the NMR tube	1	Stepper motor unit: PD60-3-1260-TMCL, PANdrive™ including NEMA24, TMCM-1260 controller/drive electronics, and TRINAMIC™ sensOstep™ encoder for step-loss detection.	1	Reichelt
	2	reference switch, Mar 1045.6502, Marquardt	1	Voelkner
	3	cables		
	4	3D-printing filament (Shuttle, PETG)	800 g	dasfilament.de
	5	USB-cables		
	6	Flexible rod, Module 636 teeth, 2000 mm (521-7837, PFR10/10/2), pitch module 1.0	1	rs-online.com
	7	Gear wheel (pitch module 1.0, with 37 teeth), quick connector (3D printed, PETG)	1	
	8	Motor cooling fan (BlackSilent Pro Fan PR-2, 60 mm)	1	Noiseblocker
	9	Motor power supply (199 W, 24 V, 8,3 A, OWA-200E-24)	1	MEAN WELL
4 - SE	1	Alu tubes 30 × 2 mm, 400 mm	1	Alu-verkauf.de
	2	1 mm isolated copper wire	~160 m	Synflex Elektro GmbH
	3	Power supply (NGL 202)	1	Rohde&Schwarz
	4	Epoxy resin 105 (Coating)	200 g	West system
	5	3D-printing filament (SE coil support, PETG)	400 g	dasfilament.de
	6	SE cooling fans (BlackSilent Pro Fan PR-2, 60 mm)	3	Noiseblocker

5 - MS	1	MS-1L (modified for 54 mm axial hole)	1	Twinleaf
	2	Low noise bipolar power supply (CSB3-40)	3	Twinleaf

The 3D printed parts were constructed using computer-aided design (CAD) and 3D printing of polylactic acid or poly(methyl methacrylate) (CAD, Inventor, Autodesk, MK3S+, Prusa; Cura slicer, Ultimaker; Formlabs). PETG is a more durable material than PLA; therefore, it was used for printing certain parts. Alternatively, one can try using nylon-based printing materials. The currently used PETG gear wheel was used to shuttle over a distance of 50 km or over 25,000 cycles without a material failure. We also tested with PLA gear wheels, but their cycle-to-break was only a few km. Since printing is not as precise as machining, we optimized the fit of the gear and rod with several iterations of the wheel. An Ultimaker printer with a fine nozzle and very slow printing speed (2-5 mm/s) was used to achieve sufficient precision and fit.

2. Reference magnetic field profiles for nine shim axes

In **Fig. 4B**, the main steps leading to the simulated shimming are shown on the left. These steps require two datasets.

One is the initial field profile inside the μ-metal chamber without using any shim coil, which must be acquired each time the optimization process has to be performed. An example of this measurement is shown in **Fig. 4A**. The second dataset is composed of the field profiles of each shim coil: X, Y, Z, dY/dx, dZ/dx, dZ/dy, dY/dy, dZ/dz, d^2Z/dz^2). This dataset needs to be acquired only once, as the setup of shim current supplies and shim coils inside the μ-shield remain unchanged.

All of the nine shim coil field profiles have been acquired in the same way. First of all, for any current under 1 mA, the magnetic field produced by any shim coil can be defined as $\boldsymbol{B} = I\boldsymbol{\mu}$, where $\boldsymbol{\mu}$ is the characteristic direction and field strength per current. Therefore, we are actually trying to determine $\boldsymbol{\mu}$ for each of the nine shim coils.

To achieve this objective, a current of 20 mA is supplied to a single shim coil, while the other 8 remain shut down. The field profile is acquired and is named $\boldsymbol{B}_+$. This field profile measurement is then reproduced, but with a negative current (-20 mA), giving what is called $\boldsymbol{B}_-$. Knowing that 20 mA is included in the linear current supply range of each shim coil, we can deduce that $\boldsymbol{B}_+ = \boldsymbol{B_b} + \boldsymbol{B}_{\mathbf{coil}} = \boldsymbol{B_b} + I_+\boldsymbol{\mu}_{\mathbf{coil}}$, and the same with negative current. In the end, we can express the magnetic field per mA for each coil as $\boldsymbol{\mu}_{\mathbf{coil}} = \frac{(\boldsymbol{B}_+ - \boldsymbol{B}_-)}{(I_+ - I_-)} = \frac{(\boldsymbol{B}_+ - \boldsymbol{B}_-)}{40}$.

These 18 measurements were performed, giving a complete magnetic field profile for each of the 9 shim coils, as shown in **Fig. S1**. Although there seems to be a discrepancy between the shim coils and the magnetometer's axes for X and Y, each shim coil appears to have the expected effect on the magnetic field along the z direction inside the sample area. Note that the sample area was chosen following these measurements to match the most homogenous region of the μ-shield.

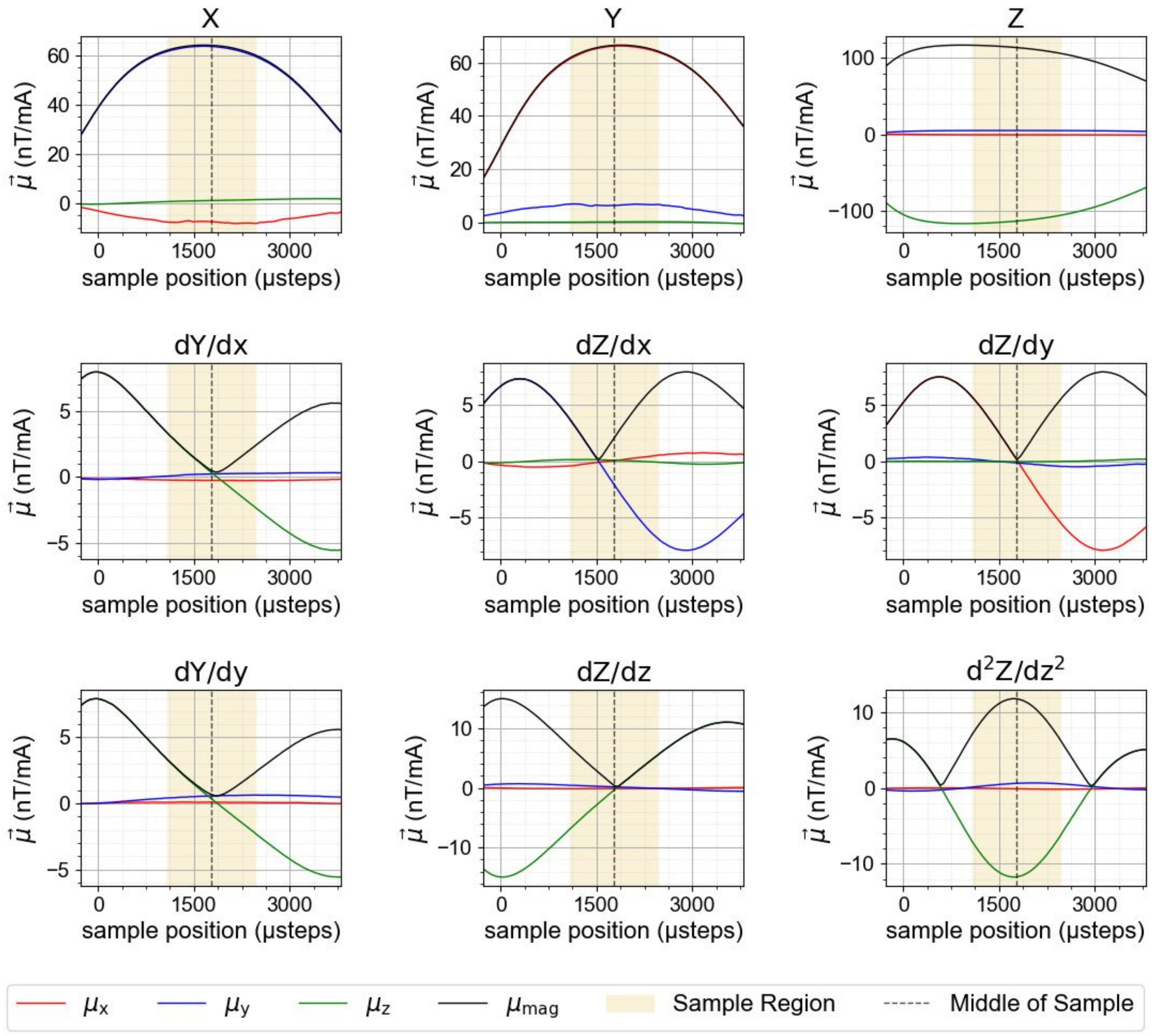

***Fig. S1: Magnetic field profile produced by each of the nine shim coils inside the µ-shield, depending on the supply current.** The measured magnetic field components as read by the magnetometer (μ_x, μ_y, μ_z) are respectively represented in red, blue, and green, while the magnitude corresponds to the black curve. The chosen 5 cm sample region is highlighted in light yellow, and the black dotted line represents its middle at 1765 µ-steps. The supply currents used as the reference for deducing these curves were ± 20 mA. The Z coil has the most influence on the overall magnetic field, with values slightly higher than 0.1 mT/mA on B_z in the sample area. The X and Y coils then follow with values slightly over 60 nT/mA. The first-order gradient coil dZ/dz has a clear linear pattern, and the second-order gradient coil d^2Z/dz^2 also has a clear quadratic pattern for μ_z in the sample region. For the last four gradients (dY/dx, dZ/dx, dZ/dy, dY/dy), the impact on the secondary for these gradients' z axis is nonlinear. Note, the sample position is shown in µ-steps used for the actual samples which differs by 2335 µ-steps from the µ-steps of the magnetometer probe. To measure such low fields, we used a magnetoresistive vector magnetometer (VMR, Twinleaf).*

3. Simulation of Z-shim coil current offsets for SABRE experiment

We selected the optimal magnetic field profile for the SABRE experiment shown in **Fig. 7** based on calibrations performed before the experiment. First, the field profile was optimized and minimized using the same process as in **Fig. 4B**. Then, knowing that the Z-shim coil (providing an offset without gradient) had the biggest impact on the field magnitude, changing its coil current was enough to reach the desired field magnitudes without affecting the field homogeneity across the sample range. For the SABRE experiment in **Fig. 7**, 37 different magnetic fields between -3 and +3 μT were selected based on the calibration described in the Methods section (main manuscript). To confirm no detrimental effects on the homogeneity of the field magnitude when applying the Z-shim offset, we performed simulations for each of the 37 selected field, each with a specific Z-shim offset in the range ± 28 mA. For this we used the final field profile acquired in **Fig. 4C** just before the SABRE experiment and added the Z-shim offsets using the simulation, extracting all the field profiles for the 37 offsets. These field profiles are shown in **Fig. S2A**, and the magnitude average and standard deviation are plotted in **Fig. S2B**.

Fig. S2A demonstrates that the magnetic field Z-component, with $|B_Z| \approx B_{mag}$ since the other two components have already been minimized, is particularly homogeneous in the sample area for B_{mag} below 1000 nT. **Fig. S2B** clearly showcases the linearity of the magnetic field magnitude depending on the Z-shim coil current offset. This indicates that using the Z-shim offset to vary the field magnitude for a SABRE field sweep was a valid approach. Also, calibrating the fields using the linear fit applied to the field measurements at the sample middle position (z_{sample}) as described in the main manuscript yielded reliable results **Fig. S2B**. For example, we decided on a 450 nT (according to the linear fit) field magnitude at z_{sample}, which yielded a 471.4 ± 9.5 nT field magnitude over the whole 5-cm sample region according to the simulation (<5% discrepancy).

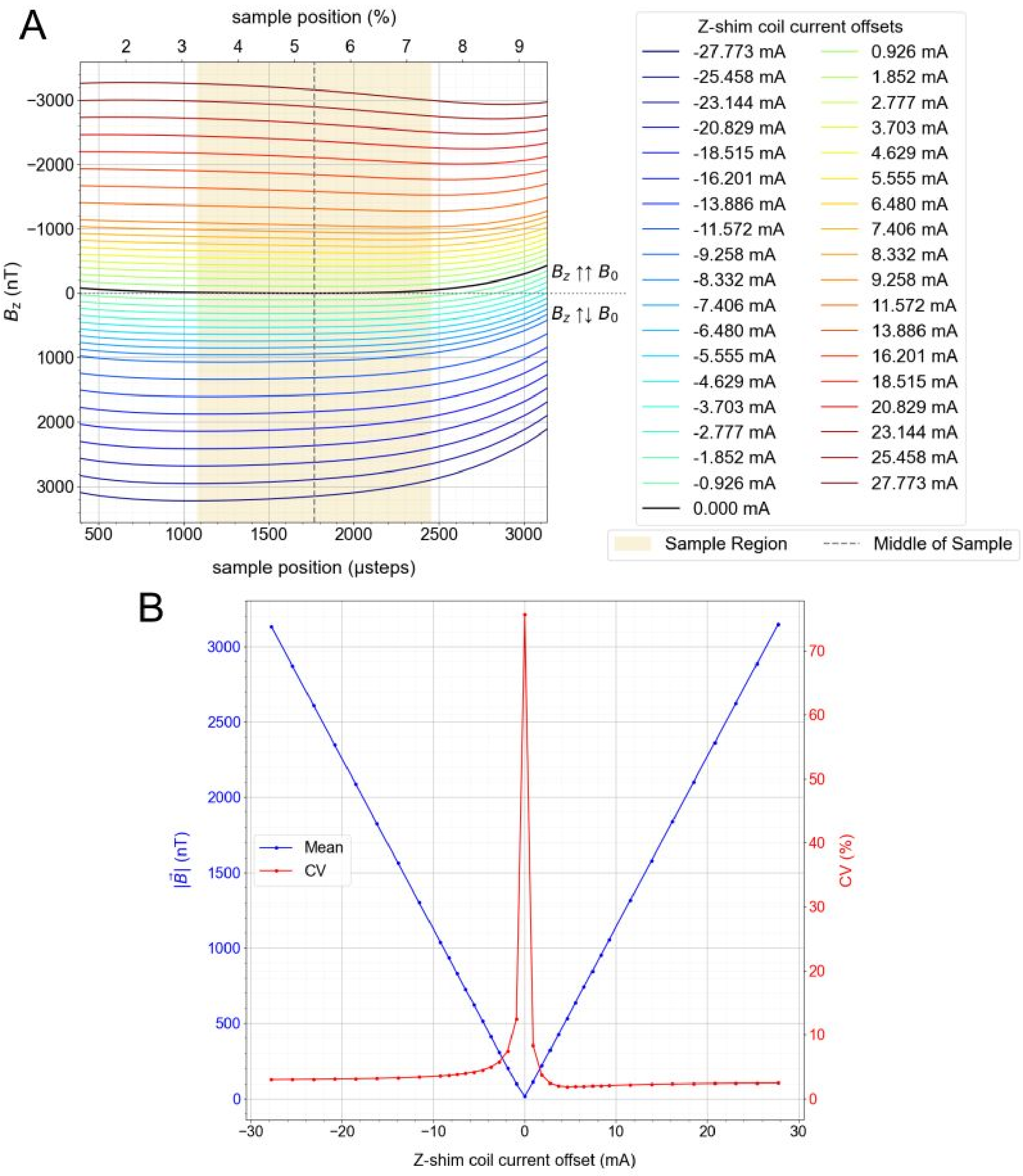

Fig S2: Simulations of the magnetic field for all 37 current offsets of the Z-shim coil in the range ± 28 mA used for the SABRE field sweep. *(A) Each simulation is represented with the magnetic field z-component over the sample area and beyond. For B_z < 0 (according to the magnetometer used), the magnetic field z-component is aligned with the magnetic field produced by the spectrometer, and vice versa. (B) The average field magnitude over the sample region (blue) and its coefficient of variation (CV, red) were extracted, and the points were connected to guide the eye. The average magnitude evolves linearly between -3000 mA and 0, as well as 0 and 3000 mA. The CV remains constant for mean magnitudes higher than approximately 250 nT (offset of ~2.3 mA). Accordingly, the CV is largest for lower averages (about 75% for the lowest field of 14.5 nT) and decreases to about 2-4% with increasing fields.*

4. Examining the effects of mispositioning the magnetometer with respect to the shim calibration

As slight imperfections and misalignment can appear over time on any frequently used scientific setup, we found it relevant to examine the effects of mispositioning the magnetometer to acquire $\boldsymbol{B}_b$ with respect to the position of the magnetometer used during the initial shim coil calibration explained in section 2 (SI).

For example, the motor could have a slight error on the vertical positioning of the magnetometer inside the µ-shield, even though the same programming instructions are being used to control it each time. For example, the slack in the flexible gear rod system allows for some variability both in vertical position (translation) and in rotation. Further, the µ-shield can be rotated by a few degrees in its mount, which changes the alignment of the X and Y coils with respect to the magnetometer axes. The impact of translation and rotation errors is worth examining on the minimized field profile, as well as the field profile used for the SABRE experiment in **Fig. 7**.

The procedure to check for these errors was follows. First, the overall field profile $\boldsymbol{B}_b$ was measured. Then, as usual, the simulative tuning (from the optimization process shown in **Fig. 4B**) was applied to minimize the field profile. When looking at the SABRE field, the desired Z-shim coil current offset was considered during this step. Additionally, for multiple position or rotation offsets on the $\boldsymbol{B}_b$, the same simulative tuning step was reapplied. For each tuning step, the theoretical average and standard deviation over the 5-cm sample length were saved. The data obtained through this technique is shown in **Fig. S3**.

Overall, while the translational and rotational error played a significant role for lower fields of a few nT (**Fig. S3A,B**). However, these effects lose significance once moving towards higher fields (**Fig. S3C,D**). Here, even a position error of 10 mm changes the average magnetic field by less than 15% for a 5 cm sample region.

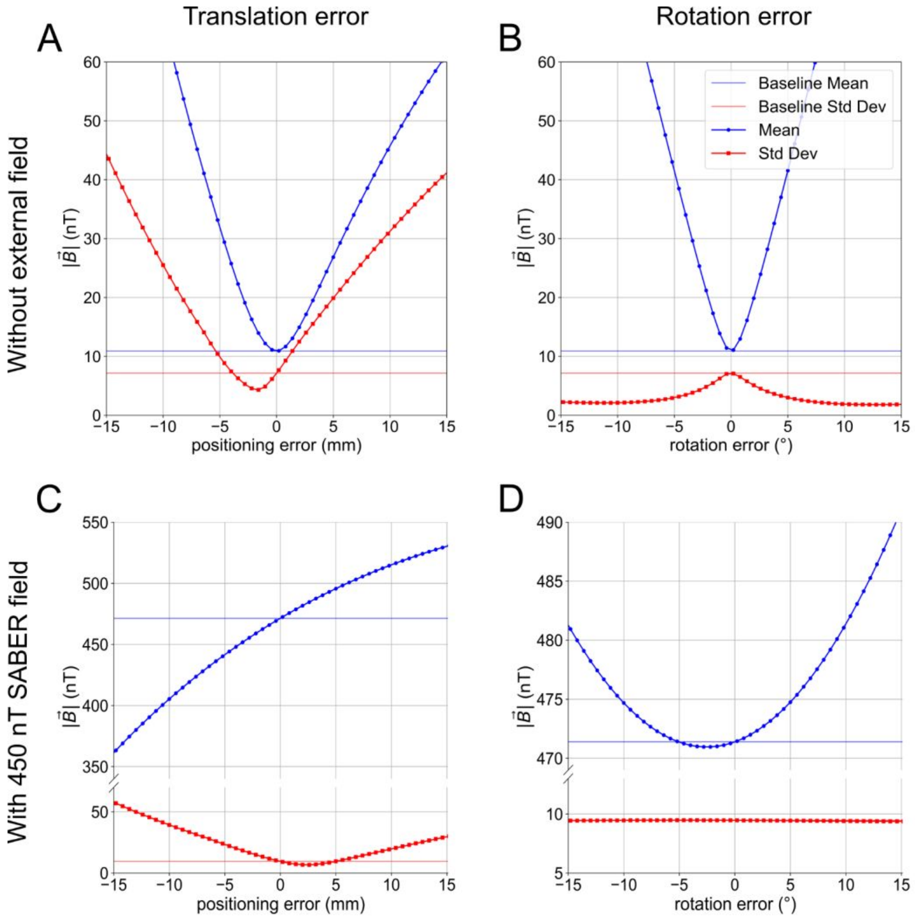

Fig. S3: Estimation of the impact, for a 5-cm sample, of an error on the magnetometer vertical position or rotation error with respect to the shim coil calibration, with or without magnetic field offset. *(A, B) Following the optimization of Fig. 4 (main manuscript) the simulated field was 10.9 ± 7.1 nT. (A) With increasing position error, the average field magnitude and its standard deviation increase. An x-offset can be observed for the standard deviation, as its minimal value was observed if the sample is positioned a few millimeters too high. This is expected, because we minimized the overall magnitude and not standard deviation. (B) A rotational error of only a few degrees induces a considerable change in the field magnitude (more than 35 nT for only 5°). Interestingly, the magnetic field gets more homogeneous with increasing rotational error. (C, D) The selected optimal magnetic field for SABRE experiments was around 450 nT at* z_{sample}*, which is estimated to represent 471.4 ± 9.5 nT across the sample range. (C) Unlike (A), the average is lower for a position error towards the top of the μ-shield and higher towards the bottom. The standard deviation, however, is increasing again about linearly with the translation error. While an offset can also be observed for the standard deviation, in this case its minimal value is expected if the magnetometer is positioned a few millimeters too low. (D) The average field magnitude increases strongly with rotational error, with its minimum at around -3º. The standard deviation remains simply constant and below 10 nT.*

5. Details about the *Python* code for magnetic field shimming

The whole shimming procedure shown in **Fig. 4B** is written with *Python* and is composed of multiple files. There are two main scripts, seven function files, and one secondary script with a unique purpose. The seven function files are named with the following keywords: CSB, data, motor, optimization, PSU, tuning, and VMR.

CSB is the model of a 3-port current supply from Twinleaf. Since the µ-shield has nine shim coils, three CSBs are included in this setup. Ultimately, the CSB functions allow to read and control each of the nine coil currents. Actually, to counter technical issues coming from the simultaneous connection of the three CSBs through separate USB ports, the CSB functions interact with each CSB through its own subprocess. Each subprocess receives its own instructions, which are defined in another *Python* file (CSB device worker). The latter communicates with the CSBs thanks to Twinleaf's TIO *Python* package.

The data functions are all important but not necessarily linked with each other. The main ones are related to weighted positions generation and data interpolation. There is also a section exclusively for defining the exact dimensions of the experimental setup, so that conversion between different units for vertical positioning is more user-friendly.

The motor, PSU and VMR functions have explicit names. Their objective is to fully control their one specific device, which are the vertical motor holding the sample or magnetometer, the solenoid-coil used for MFC, and the Twinleaf's vector magnetometer probe, respectively.

Finally, the simulative tuning and iterative tuning each have a dedicated function file.

The first main script is used for the initial calibration of the nine shim coils. Its purpose is described in section 2 (Supporting Information). There is no need to use it more than once if the experimental setup remains the same afterwards. A full calibration of all shims takes about 2-3 hours for an acceptable vertical position resolution.

The second main script is the actual complete shimming procedure, from A to Z. It is divided into seven steps: initial magnetic field profile measurement, simulative tuning, applying new currents, second magnetic field profile measurement, iterative tuning, applying new currents, final magnetic field profile measurement and comparison. The user can also change a few parameters beforehand, such as the use of the PSU to generate a much higher ambient field using the solenoid-coil before each field profile measurement.

6. Raw data of Fig. 5:

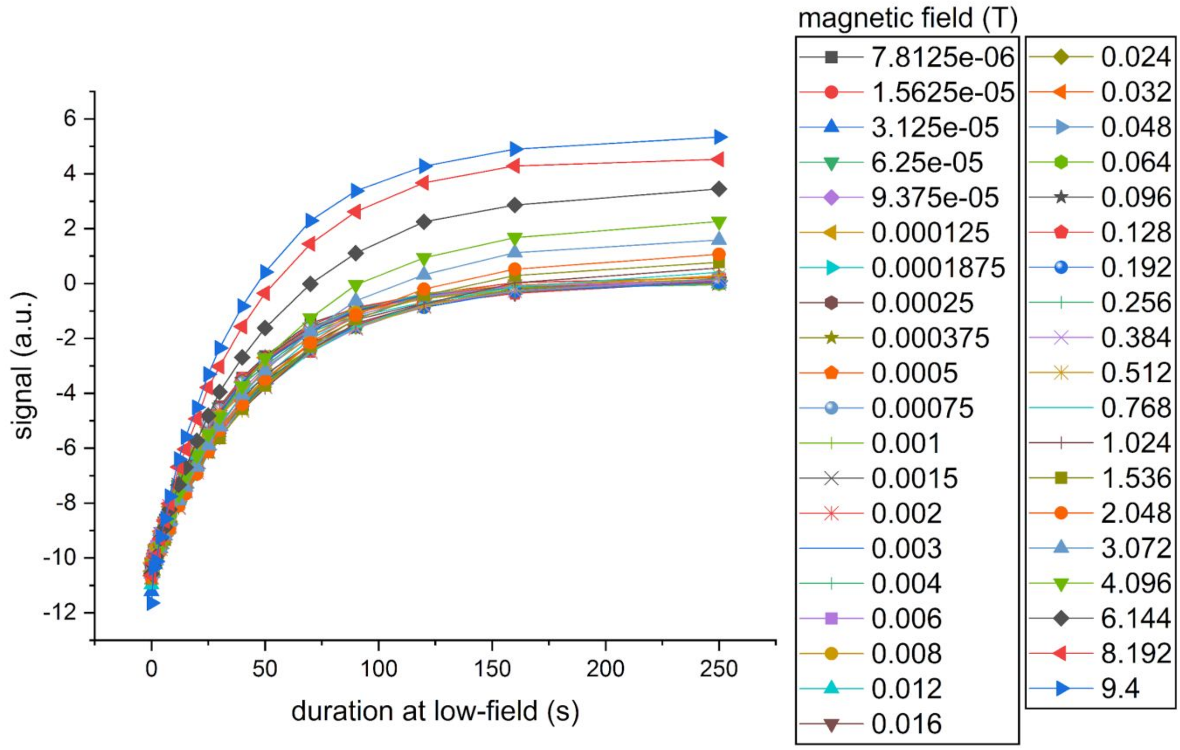

Fig. S4: NMR magnetic field dispersion (NMRD) curves of the DNP sample for all 39 fields between 7.8 μT and 9.4 T. The 19 measured points per NMRD curve were connected with lines to guide the eye.

Tab. S2. Extracted fitting data to the function $y = A_1 \cdot expexp\left(-\frac{t}{T_1}\right) + A_2 \cdot expexp\left(-\frac{t}{T_{1_2}}\right) + y_0$, with T_{12} being shared across all experiment with (3.70±0.34) s.

field (T)	T_1 (s)	T_1 stdev (s)	R_1 (1/s)	A_1 (a.u.)	A_1 stdev (a.u.)	y_0 (a.u.)	y_0 stdev (a.u.)	A_2 (a.u.)	A_2 stdev (a.u.)
7.8125E-06	36.934	0.73677	0.027075324	-101556.3	803.7115	98.37075	498.6711	-159.515	1205.973
1.5625E-05	39.98259	0.8153	0.025010886	-101587.7	803.1228	2045.946	525.7138	-6079.2	1179.241
0.00003125	41.24206	0.92543	0.024247091	-97570.69	886.2854	975.019	541.4955	-12757.7	1211.599
0.0000625	37.9659	0.75919	0.026339426	-101712.5	796.5497	255.1632	507.4325	-2272.89	1193.194
0.00009375	38.3225	0.7675	0.026094331	-101430.2	788.7323	262.4671	510.2904	-1025.22	1186.243
0.000125	40.33948	0.82191	0.024789611	-100517.4	776.2063	1450.408	527.7905	-3073.64	1163.138
0.0001875	39.27771	0.78563	0.025459733	-101832.4	780.4076	782.0183	518.4374	-1654.95	1174.001

0.00025	38.2199	0.75615	0.02616438	-102608.1	788.8609	597.9257	509.3923	86.0931	1187.201
0.000375	39.21775	0.78884	0.025498658	-101249.9	781.0036	270.2265	517.9267	-1655.11	1174.797
0.0005	40.49881	0.81602	0.024692084	-101387.8	769.2565	1439.336	528.9294	-1737.29	1158.484
0.00075	38.38749	0.7699	0.026050153	-101284.9	787.7765	-13.2198	510.8283	-881.258	1185.211
0.001	39.81306	0.80192	0.025117386	-101446.7	779.1302	638.4906	523.1727	-2674.14	1168.895
0.0015	39.7133	0.7993	0.025180481	-101303.8	776.3025	429.0162	522.163	-1694.52	1168.357
0.002	39.84892	0.80107	0.025094783	-101536.6	776.5479	680.8688	523.3883	-2145.15	1167.347
0.003	39.89953	0.80085	0.025062952	-101558.8	773.2579	590.8109	523.7066	-1184.7	1165.338
0.004	40.27132	0.80585	0.024831567	-101999.2	770.6323	-67.2384	526.9372	-1508.41	1161.006
0.006	43.8741	0.9138	0.02279249	-99930.17	758.9951	1464.732	559.5805	-4758.65	1127.267
0.008	43.55267	0.92367	0.022960705	-99520.64	786.7181	752.5292	558.1461	-7583.6	1143.12
0.012	44.88813	0.94104	0.022277604	-100940.8	773.9049	549.1476	570.1709	-7289.83	1127.003
0.016	45.26352	0.91761	0.022092847	-102794.9	744.7903	425.2799	571.896	-3798.42	1110.269
0.024	46.50097	0.93467	0.021504928	-103712.2	732.0427	678.37	582.8931	-2113.05	1095.363
0.032	46.16348	0.92885	0.021662145	-103426	732.8599	568.6386	579.7096	-1876.3	1098.149
0.048	47.61265	0.96448	0.021002822	-103782.4	733.5425	937.7242	593.775	-3678.29	1088.269
0.064	46.87656	0.94601	0.021332623	-103608.3	732.7936	308.7475	586.5689	-2816.41	1093.063
0.096	48.3698	0.96584	0.020674057	-105155.4	724.603	1145.686	600.4643	-1829.78	1078.804
0.128	49.68216	1.02934	0.020127949	-102924.7	734.2963	1197.152	614.2846	-5090.54	1074.488
0.192	48.27809	0.97896	0.02071333	-103836.4	729.4049	647.4021	599.957	-3261.29	1081.707
0.256	49.00603	0.98124	0.020405652	-105149.6	722.7117	1447.628	606.5498	-1778.19	1073.553
0.384	51.06347	1.04965	0.019583471	-104060.7	726.7991	1907.846	627.4296	-4258.06	1061.667

0.512	52.42661	1.0723	0.019074283	-105627.7	728.9444	3345.209	641.5028	-5105.35	1053.725
0.768	52.57502	1.04264	0.01902044	-108170.2	719.2995	4215.545	641.9167	-2729.75	1048.087
1.024	53.90524	1.06458	0.018551072	-109371.8	718.9282	5870.762	655.4398	-2760.7	1039.206
1.536	55.64213	1.09424	0.017971994	-111677.7	727.4907	8938.416	674.5724	-5033.75	1032.165
2.048	54.70185	1.02805	0.018280917	-115397.6	718.9126	11337.76	663.6191	-2690.81	1034.013
3.072	55.21381	0.99865	0.018111411	-120294.7	719.6991	17257.35	669.0065	-2893.23	1031.074
4.096	55.14725	0.96787	0.01813327	-124958.5	728.6692	23643.27	669.5791	-5326.37	1035.943
6.144	50.34636	0.78285	0.019862409	-136976.8	727.0006	34772.12	620.2446	-3995.09	1066.54
8.192	44.53852	0.61519	0.022452475	-150100.7	744.6965	46275.31	565.0068	-2945.8	1115.523
9.4	43.17757	0.60956	0.023160173	-154822.9	851.6385	53145.06	558.3838	-12059.4	1178.627
